# BTK inhibitor ibrutinib reduces LPS-induced inflammation in C8-B4 microglia

**DOI:** 10.17179/excli2025-8695

**Published:** 2025-11-05

**Authors:** Debanjan Das, Akash S. Mali, Denise Greco, Danica Michalicková, Jirí Novotný, Ondrej Slanar

**Affiliations:** 1 Institute of Pharmacology, First Faculty of Medicine, Charles University and General University Hospital, Prague, Czech Republic; 2 Department of Physiology, Faculty of Science, Charles University, Prague, Czech Republic; 3 A. I. Virtanen Institute for Molecular Sciences, University of Eastern Finland, Kuopio, Finland

**Keywords:** ibrutinib, lipopolysaccharide, microglia, oxidative stress, Nrf2, NF-kappa beta

## Abstract

In this study, we examined the potential of Bruton tyrosine kinase (BTK) inhibitor ibrutinib to mitigate neuroinflammation in C8-B4 microglial cells activated by the bacterial endotoxin lipopolysaccharide (LPS). Our objective was to enhance understanding of its mechanism of action, particularly in relation to its anti-inflammatory, and antioxidant potential of ibrutinib. Here, mouse microglial C8-B4 cells were treated with ibrutinib (1 and 10 μM) or vehicle (1 % DMSO) for 1 h, followed by lipopolysaccharide (LPS 1 μg/mL) for 23 h. We observed that ibrutinib significantly decreased LPS-induced nitric oxide levels and nitric oxide synthase 3 (NOS3) expression. In parallel, ibrutinib decreased cell senescence induced by LPS in microglia. Ibrutinib notably diminished the elevation of tumor necrosis factor-α (TNF-α), triggered by LPS in C8-B4 microglia. It also modulated Toll-like receptor 4 (TLR4) expression induced by LPS. Moreover, ibrutinib markedly lowered the augmented levels of nuclear factor kappa beta (NF-κβ) and phosphorylated NF-kβ (pNF-κβ) induced by LPS, indicating its capacity to mitigate LPS-induced neuroinflammatory reactions by hindering TLR4/NF-κβ pathway. Additionally, these beneficial effects are associated with regulation of the Nrf2/HO-1 pathway. The present results suggest that treatment with ibrutinib may contribute to the preservation of mitochondrial function, as evidenced by its ability to reduce reactive oxygen species (ROS) production. While these findings provide important insights into the potential neuroprotective mechanisms of ibrutinib, the precise molecular pathways involved in mitochondrial preservation require further investigation. Collectively, these data support the therapeutic potential of ibrutinib in mitigating neuroinflammation-related mitochondrial dysfunction and highlight its promise as a candidate for treating neurodegenerative disorders characterized by oxidative stress and impaired mitochondrial integrity.

See also the graphical abstract(Fig. 1).

## Background

The neuroimmune system plays a role in multiple functions of the central nervous system (CNS), including development, aging, and response to injury. Microglia are the main cells responsible for the first line of defense against external toxic substances and proinflammatory reactions in the normal brain (Hines et al., 2009[12]). Additionally, microglia are involved in synaptic development and neurogenesis, a process of forming new neurons from neuronal stem cells and a major event in the development of the nervous system (Sierra et al., 2010[34]; Paolicelli et al., 2011[30]; Schafer et al., 2012[33]). Microglia constitute 5-12 % of the overall CNS cell population. Upon activation by pathologic stimuli, microglia change their morphology to a rod-like shape and upregulate the secretion of neuroinflammatory mediators, such as tumor necrosis factor α (TNF-α) (Kirkley et al., 2017[17]; Yin et al., 2018[42]). These neuroinflammatory responses are strongly associated with neurodegenerative diseases like multiple sclerosis (Michaličková et al., 2022[25]), Alzheimer's disease (AD), stroke, cognitive dysfunction, neuronal cell death, and synaptic degeneration (Hirsch and Hunot, 2009[13]; Saijo et al., 2009[32]). Therefore, controlling the neuroinflammatory response holds potential as a therapeutic approach for diseases related to neuroinflammation and neurodegeneration. 

Toll-like receptors (TLRs) are a class of receptors that are activated by detection of foreign elements, for example lipopolysaccharide (LPS), and start the downstream signaling cascade that regulate the gene transcription of pro-inflammatory mediators (Warren et al., 2010[38]). LPS activation of TLR4 transmembrane protein influence microglia polarization and directly influences sensory neurons (Acosta and Davies, 2008[1]; Durafourt et al., 2012[7]; Catorce and Gevorkian, 2016[2]). Microglia polarization is characterized by the escalation of pro-inflammatory factors and reactive oxygen species (ROS). ROS are key factors associated with M1 microglia polarization, and their excessive production can lead to sustained damage to the brain (Tan et al., 2016[36]; Tang and Le, 2016[37]; Zhang et al., 2021[43]). Another important factor in cellular resistance to oxidants is the nuclear transcription factor erythroid 2 related factor-2 (Nrf2) (Ma, 2013[21]), which is a critical transcription factor that regulates the expression of antioxidant and phase II detoxification genes. Under normal conditions, Nrf2 is kept in the cytoplasm by its inhibitor, Keap1. However, oxidative stress or other stimuli lead to the dissociation of this complex, allowing Nrf2 to translocate to the nucleus where it activates genes that counteract oxidative damage and inflammation (Michaličková et al., 2020[24]). Heme oxygenase-1 (HO-1), a key downstream enzyme regulated by Nrf2, plays a crucial role in anti-inflammatory activity in the brain (Khan et al., 2011[15]; Farrell-Dillon and Fraser, 2016[9]; Loboda et al., 2016[20]). Nrf2 also controls superoxide dismutase (SOD) and catalase, enzymes responsible for neutralization of the superoxide anion and hydrogen peroxide, respectively.

Bruton tyrosine kinase (BTK) is a segment of non-receptor associated TEC tyrosine kinase family and can be found in almost all hematopoietic cell types, with the exception of plasma cells and T cells (Das et al., 2025[4]). Its role involves regulating immune response signaling through B cell receptor (BCR), ultimately resulting in B cell activation, differentiation and chemotaxis (Niiro and Clark, 2002[27]). Besides being essential for B cell development, BTK is also involved in TLR signaling within B cells (Mangla et al., 2004[23]; Rip et al., 2019[31]). Consequently, inhibiting BTK can result in decreased inflammation in the CNS. At present, a first generation BTK inhibitor (BTKi) ibrutinib and two second generation BTKi acalabrutinib and zanubrutinib are approved by the Food and Drug Administration (FDA) (de Claro et al., 2015[6]; Kim and Prasad, 2020[16]; Wen et al., 2021[39]). Ibrutinib, acting as an immunomodulator, affects the function of B cells and macrophages. It also plays a role in the phosphorylation of TLR signaling pathways, which are associated with the production of pro-inflammatory cytokines. Ibrutinib has been found to reduce the production of pro-inflammatory cytokines IL-6 and TNF-α in the patients with systemic sclerosis (Einhaus et al., 2020[8]). Moreover, it has been observed that ibrutinib lowers the levels of pro-inflammatory cytokines triggered by LPS in microglial cells by inhibiting the TLR4/NF-κβ signaling pathway (Nam et al., 2018[26]). However, the exact role of ibrutinib in reducing oxidative stress in microglia remains uncertain. In this study, we investigated the potential of ibrutinib to reduce neuroinflammation and enhance antioxidant activity in C8-B4 microglial cells activated by the bacterial endotoxin LPS. Our goal was to gain a deeper understanding of its mechanism of action, especially concerning its anti-inflammatory, antioxidative, and cytoprotective properties.

## Materials and Methods

### Drugs, chemicals and antibodies

C8-B4 cells were purchased from the American Type Culture Collection (CRL-2540™, Rockville, MD, USA). Lipopolysaccharide (L2637; E coli 055:B5) was obtained from Sigma-Aldrich (St. Louis, MO, USA). Ibrutinib (CAS number 936563-96-1, purity ˃ 99 %) was a generous gift from Zentiva, Prague, Czech Republic. Fetal bovine serum (FBS), cell culture media were obtained from Thermo Fisher Scientific (Waltham, MA, USA). Other reagents were purchased from Merck KGaA (Darmstadt, Germany) and were available in high-analytical grade for research purpose. 

### Cell culture and treatment

C8-B4 cells were maintained in high-glucose Dulbecso's modified Eagle's medium (DMEM) supplemented with 10 % fetal bovine serum, 100 U/mL penicillin, and 100 U/mL streptomycin in a humidified environment at 37°C with 5 % CO_2_. For checking the anti-inflammatory effect of ibrutinib, C8-B4 cells were treated with ibrutinib (1 and 10 μM) or vehicle (1 % DMSO) for 1 h, followed by treatment with LPS (1 μg/mL) or phosphate-buffered saline (PBS) for 23 h and several time dependent manner like 6 h/9 h for western blot attached as an additional supplementary file (Figure S1). The LPS dose was chosen based on previously published experiments using the same cell line (Mali and Novotny, 2022[22]). 

### Cell viability assay

Cell viability was determined using the tetrazolium salt 3-(4,5-dimethylthiazol-2-yl)-2,5-diphenyltetrazolium bromide (MTT) assay. C8-B4 microglial cells were seeded in 96-well plates at a density of 4×10^4^ cells per well and incubated in culture medium to allow cell adhesion. 24 h after pretreatment with various concentrations of ibrutinib (1, 5, 10, 20, 25, 50, 100, 200 μM), MTT was added to each well, and the cells were incubated at 37 °C for 3 h. The culture medium was discarded and 50 μL DMSO was added to dissolve the MTT-formazan crystals. Absorbance was measured at 570 nm against a blank using a microplate reader (BioTek synergy HT, Agilent, Santa Clara, CA, US).

### Lactate dehydrogenase (LDH) assay

The levels of LDH in the cell supernatant were determined using an LDH Release Assay Kit (ab65391, Abcam, USA) to evaluate cell membrane integrity. To assess the maximum release of LDH, cells were incubated in pyruvate-free DMEM with LPS and ibrutinib for 24 hours, followed by a 30-minute treatment with the LDH reaction mixture before measurement. The percentage of released LDH was calculated using the following formula: releasing percentage ( %) = (OD_490nm_ test sample - OD_490nm_ low control) / (OD_490nm_ high control - OD_490nm_ low control) × 100 %.

### Nitric oxide release assay

The Griess reaction was used to measure nitric oxide (NO) production by microglial cultures. After pretreatment of C8-B4 cells with LPS and ibrutinib for 24 h, 50 μL of cell culture medium was collected and mixed with an equal volume of Griess reagent (0.1 % N-1-naphthylethylenediamine dihydrochloride and 1 % sulfanilamide in 5 % phosphoric acid) in a 96-well plate, followed by incubation for 5-10 min at the room temperature with continuous shaking in dark. The absorbance was then measured at 540 nm using a microplate reader (BioTek synergy HT, Agilent, Santa Clara, CA, US).

### Detection of intracellular reactive oxygen species generation

Detection of ROS was performed by 2',7'-dichlorofluorescin diacetate (DCF-DA, 35845, Sigma Aldrich, USA), a fluorogenic probe that is permeable to the cell membrane. C8-B4 cells were seeded into 12-well plates and cultured until they reached 80-90 % confluence. The cells then were treated with different concentrations of ibrutinib with or without LPS to observe ROS generation. The original medium was removed, and the cells were incubated with 10 μM DCF-DA in fresh medium at 37°C for 30 min. To quantify intracellular ROS, DCF-stained cells that accumulated due to the oxidation of DCF were observed under a fluorescence microscope and photographed at 10× magnification and also assayed by the flow cytometry (BD LSRII, BD Biosciences, NJ, US) with excitation wavelength at 488 nm and emission wavelength at 525 nm, respectively. ImageJ v2.14 was used to quantify the fluorescence intensity of microscopic image and FlowJo v10.10 was used to quantify flow cytometry data.

### Wound-healing assay

For the scratch wound healing assay, C8-B4 microglial cells were seeded in 12-well plates and incubated until the cells reached 80-90 % confluence. The cells were scratched with a 1 mL sterile pipette tip to create a wound. Images were captured at 0 h. Next, the cells were treated with ibrutinib (1 and 10 μM) or vehicle (1 % DMSO) for 1 h, followed by LPS (1 μg/mL) or PBS for 23 h to allow time for migration to the cell-free area. Images of scratched cell monolayers were taken at 5x magnification at baseline (0 h time point) and after 24 h of incubation. Scratch widths were measured using ImageJ software (ImageJ macro; https://github.com/AlejandraArnedo/Wound-healing-size-tool). 

### Analysis of cellular senescence

Microglia cell senescence was assessed using the senescence cells histochemical staining kit (Sigma Aldrich, CS0030). Briefly, cells were washed with Dulbecco's phosphate-buffered saline (DPBS), fixed with 1× fixation buffer and stained with 2 mL of staining mixture. Afterwards, cells underwent washing and exposed to a staining mixture at 37 °C for 24 h and observed the next day. Galactosidase beta 1 (GLB-1) - positive cells associated with senescence form a bright blue pigment in the cell that can be observed under a light microscope with 10x magnification (Leica DMI 3000B Wetzlar, Germany) and counted. 

### Cell cycle analysis

C8-B4 microglial cells were seeded in 6-well plates at a density of 1×10^6^ cells/well and cultured at 37 °C in a humidified 5 % CO_2 _atmosphere until the cell confluency reached 60-70 %. Subsequently, cells were treated after drug free synchronization by serum starvation with ibrutinib at the desired concentration (1 and 10 μM) or vehicle (1 % DMSO) for 1 h, followed by LPS (1 μg/mL) or PBS for 23 h. C8B4 microglial cells were the washed with precooled PBS twice and then fixed in 75 % ethanol overnight at 4 °C. The fixed cells were again washed twice with precooled PBS and were then stained with propidium iodide (P4170, Sigma Aldrich, USA)/RnaseA and incubated at 37°C in the dark for 30 min. The DNA content was assessed by using a flow cytometer (BD LSRII) and the data was analyzed by using FlowJo v10.10 software (BD Life Sciences, OR, US).

### Western blot

Cells were collected in ice-cold PBS (pH 7.4), pelleted by centrifugation at 1000× g (Hettich Universal 320R, Andreas Hettich GmbH & Co. KG, Tuttlingen, Germany), and resuspended in TMES buffer supplemented with cOmplete and PhosSTOP inhibitors (Roche, Basel, Switzerland). Cell disruption was achieved by sonication (Bandelin electronic GmbH & Co. KG, Berlin, Germany) for 10 seconds at 50 % amplitude. Protein concentrations were quantified using the bicinchoninic acid (BCA) assay with bovine serum albumin as a reference standard. Equal protein amounts were combined with Laemmli buffer, heated for 2 minutes, and subsequently loaded onto polyacrylamide gels (Bio-Rad, Hercules, CA, USA).

Electrophoresis was carried out at a constant voltage of 200 V for about 60 minutes, until the tracking dye reached the bottom of the 50-mm gel. Separated proteins were transferred to nitrocellulose membranes (0.45 µm pore size) and blocked for 1 hour at room temperature in 5 % skim milk prepared in TBS-T buffer (10 mM Tris, 150 mM NaCl, 1 % Tween 20; pH 8.0). Membranes were incubated overnight at 4 °C with primary antibodies directed against NOS3, TLR4, NF-κB, phospho-NF-κB, CD86, catalase, cytochrome c, and Nrf2 (Santa Cruz Biotechnology, Dallas, TX, USA), as well as against SOD3 and HO-1 (ABclonal Technology, Düsseldorf, Germany), at 1:10,000 dilution or as specified by the manufacturer. Secondary antibodies included anti-mouse (NA931V) and anti-rabbit (NA934V) IgG (GE Healthcare, Chicago, IL, USA). Blots were incubated with HRP-conjugated secondary antibodies (anti-mouse/anti-rabbit/anti-goat) at room temperature for 1 hour following TBS-T washes.

Protein detection was performed using Super Signal chemiluminescent substrates (Pierce Biotechnology, Rockford, IL, USA) and developed on CP-BU X-ray film (Agfa, Mortsel, Belgium). Membranes were subsequently reprobed with alternative antibodies after stripping with western blot stripping buffer (ab282569, Abcam Inc., MA, USA) to ensure uniform antibody removal. Band intensities were quantified using ImageJ v2.14 software (NIH, Maryland, USA), normalized to β-actin, and presented as fold changes relative to control or LPS-treated samples.

### Statistical analysis

All experiments were performed in at least three independent biological replicates. Statistical analyses were performed using GraphPad Prism software version 9.0 (GraphPad Software, San Diego, CA, US). All data were expressed as mean ± standard deviation (SD). Differences between the groups were analyzed by one-way analysis ANOVA followed by Tukey's multiple comparison post-hoc test, unless stated otherwise. *p *values of less than 0.05 were considered statistically significant.

## Results

### Effects of ibrutinib on microglia viability

**Figure 2:**
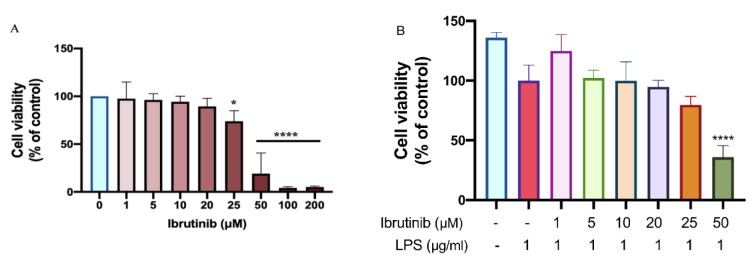
Effect of ibrutinib on viability of C8-B4 microglial cells. C8-B4 cells were treated with different concentrations of ibrutinib, and cell viability was determined after 24 h using MTT assay without LPS (A) and with LPS (B). Data are expressed as mean ± standard deviation (SD) of three independent experiments performed in triplicate. (^*^P<0.05, ^**^P<0.01,^****^P<0.0001 indicate that a group is significantly different from the control group)

To investigate whether ibrutinib mitigates LPS-induced oxidative stress and inflammation, we first examined its impact on the viability of C8-B4 microglial cells. A series of cell viability assays were performed, exposing cells to increasing concentrations of ibrutinib. The results demonstrated a dose-dependent decrease in cell viability; however, no significant cytotoxicity was observed at concentrations up to 25 μM, as shown in Figure 2(Fig. 2). Importantly, co-administration of ibrutinib and LPS did not result in additional cytotoxic effects, indicating that ibrutinib remained safe for microglia under inflammatory conditions (Figure 2B(Fig. 2)). Based on these findings, we selected two concentrations of ibrutinib: 1 μM, representing a lower, non-toxic dose, and 10 μM, representing a higher, yet still safe dose-for subsequent experiments involving LPS co-treatment. This approach allowed us to comprehensively evaluate the protective effects of ibrutinib across a physiologically relevant dose range.

### Ibrutinib prevents LPS-induced cytotoxicity in microglia

The results showed that treatment with LPS alone led to a marked increase in LDH levels, reflecting significant cellular injury and loss of membrane integrity-a hallmark of cytotoxic stress and inflammation in microglia. However, when cells were pre-treated with ibrutinib prior to LPS exposure, there was a reduction in LDH release compared to the LPS-only group. This reduction in extracellular LDH suggests that ibrutinib confers a protective effect, minimizing membrane damage and helping to preserve microglial cell viability even under inflammatory conditions.

**Figure 3:**
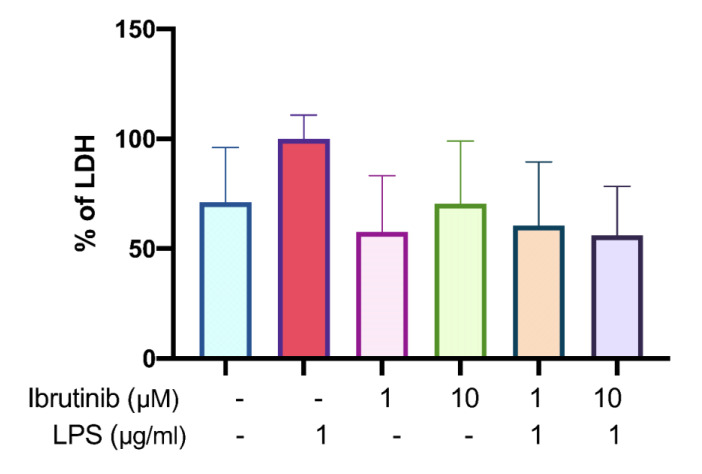
LPS-induced cytotoxicity in C8-B4 microglia cells. Secreted LDH level after 24h treatment with ibrutinib with LPS. Data are expressed as mean ± standard deviation (SD) of three independent experiments performed in triplicate.

These findings support the conclusion that ibrutinib mitigates the toxic effect of LPS on microglial cells, likely by stabilizing cellular membranes and alleviating LPS-induced cytotoxicity. The decrease in LDH release provides functional evidence that ibrutinib can suppress or counteract the damaging inflammatory response initiated by LPS. As shown in Figure 3(Fig. 3), this protective role of ibrutinib could be important for preserving microglial function during neuroinflammatory challenges.

### Ibrutinib inhibits NO production in microglial cells

**Figure 4:**
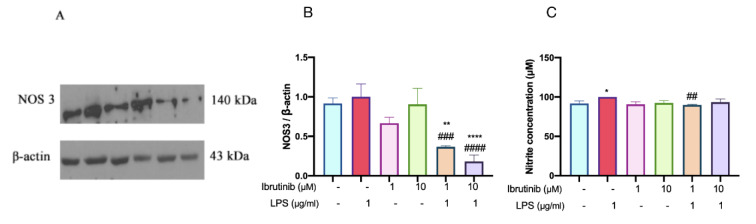
Effects of ibrutinib on NO production on microglial C8-B4 cells. C8-B4 cells pre-treated with ibrutinib for 1h prior to LPS for 23h. NOS3 protein measurement by western blot (A&B) and conditioned media were collected for nitrite measurement (C) by griess reagent. Data are expressed as mean ± standard deviation (SD) of three independent experiments. ^**^P<0.01 indicates a group is significantly different from control group and ^#^P<0.05, ^##^P<0.01,^ ###^P<0.001, ^####^P<0.0001 indicate a group is significantly different from the LPS group.

After 24-hour incubation with both LPS and ibrutinib, western blot analysis (Figure 4A and 4B(Fig. 4)) revealed that LPS treatment markedly upregulated the protein expression levels of endothelial nitric oxide synthase (NOS3) in microglial cells. Treatment with ibrutinib at both concentrations (1 μM and 10 μM) led to a notable reduction in NOS3 protein expression compared to the LPS-only group. The inhibition was observed in a dose-dependent manner, where 10 μM achieved a slightly greater reduction than 1 μM. This suggests that ibrutinib effectively suppresses LPS-induced NOS3 upregulation, which could in turn attenuate excessive NO production and mitigate associated inflammatory and cytotoxic effects.

Consistent with the increase in NOS3 expression, LPS stimulation significantly elevated NO production, as measured in the cell culture supernatant. Elevated NO is a hallmark of microglial activation and is associated with oxidative and nitrosative stress leading to neuronal damage. Notably, pre-treatment with ibrutinib at both 1 μM and 10 μM concentrations substantially reduced NO levels compared to LPS alone. The reduction in NO was statistically significant in both dosing groups, supporting the potential of ibrutinib to counteract LPS-induced detrimental effects in microglia.

Representative original western blots of NOS3, along with all other mediators assessed by this method, are presented in the supplementary information (Figure S2).

### Wound healing effect of the ibrutinib on LPS-stimulated C8-B4 microglial cells

**Figure 5:**
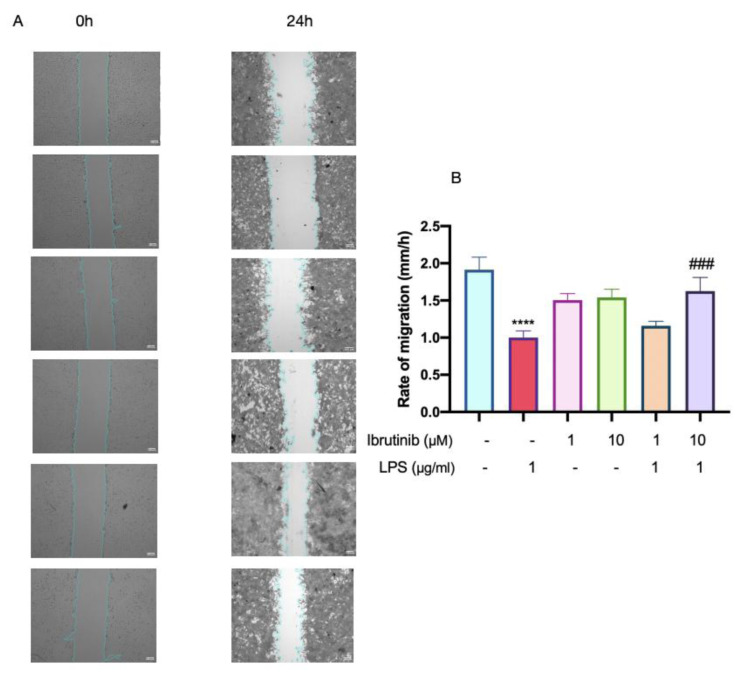
Effect of ibrutinib on the migration of mouse microglial C8-B4 cells. Data are expressed as mean ± standard deviation (SD) of three independent experiments.^ ***^P<0.001 indicates that the cell group is significantly different from the control group and ^###^*P*<0.001 indicates that a group is significantly different from the LPS group.

The migration of C8-B4 microglial cells was evaluated to determine the effect of ibrutinib on LPS-induced cellular motility, which is an important feature of microglial activation and neuroinflammation. As shown in Figure 5(Fig. 5), treatment with LPS significantly enhanced microglial migration compared to untreated control cells, demonstrating the pro-migratory effect of inflammatory stimulation. However, pre-treatment with 10 μM ibrutinib markedly inhibited this LPS-induced increase in cell migration, restoring migration rates close to baseline levels observed in control cells. This result indicates that ibrutinib effectively suppresses the migratory response elicited by LPS, thereby potentially reducing microglial-mediated neuroinflammatory damage.

### Ibrutinib prevents LPS-induced oxidative stress in microglia

**Figure 6:**
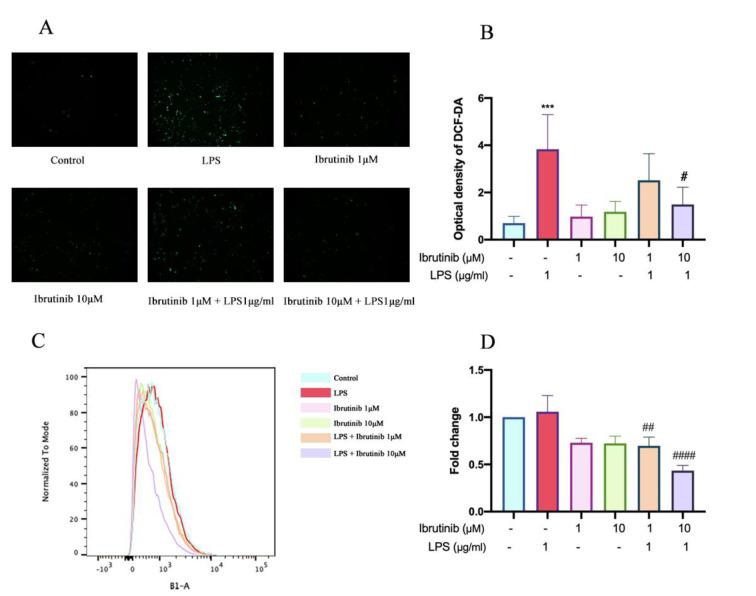
Ibrutinib prevents LPS-induced oxidative stress in mouse microglial cells. C8-B4 cells were pretreated with ibrutinib (1 and 10 μM) for 1 h and then stimulated with LPS (1μg/mL) for additional 23 h. Production of intracellular ROS was determined by DCF-DA staining using flow cytometry (A) and by flow cytometry (C) where both analysis (B and D). Data are expressed as mean ± standard deviation (SD) of three independent experiments. ^##^P < 0.01, ^####^P<0.0001 indicate that the cell group is significantly different from the LPS group.

ROS production was measured to assess the oxidative stress response of microglial cells following LPS stimulation and ibrutinib treatment. As illustrated in Figure 6(Fig. 6), exposure to LPS significantly increased intracellular ROS levels compared to untreated control cells, reflecting enhanced oxidative stress commonly associated with microglial activation and neuroinflammation. Importantly, cells pretreated with ibrutinib at the tested concentrations showed a marked and statistically significant reduction in ROS production relative to the LPS-only group. This suggests that ibrutinib exerts potent antioxidant effects, effectively counteracting the oxidative stress induced by LPS. These results support the role of ibrutinib as a modulator of both inflammatory and oxidative pathways in microglia, contributing to its potential neuroprotective properties.

### Ibrutinib reverses LPS - induced senescence in microglial cells 

**Figure 7:**
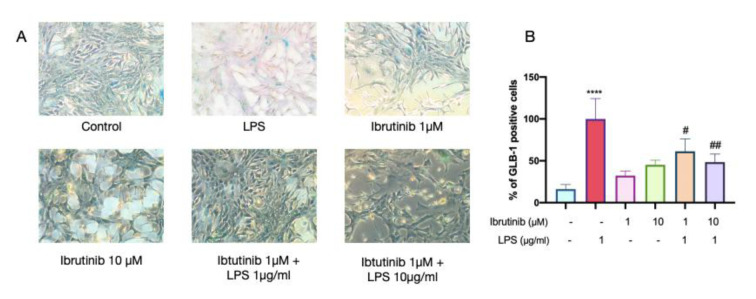
Ibrutinib prevents LPS-induced senescence-associated GLB-1 activity in mouse microglial cells. C8-B4 cells were pretreated with ibrutinib (1 and 10 μM) for 1h and then stimulated with LPS (1μg/mL) for an additional 23 h. Cellular senescence was determined by senescence staining for 72h by using confocal microscopy (A) and its statistical analysis (B). Data are expressed as mean ± standard deviation (SD) of three independent experiments. ^****^P<0.0001 indicates that the cell group is significantly different from control group and ^#^P<0.05, ^##^P<0.01 indicates that the cell group is significantly different from LPS group.

The proportion of C8-B4 microglial cells positive for senescence-associated β-galactosidase (GLB-1) staining was significantly increased following LPS treatment (Figure 7B(Fig. 7)), indicating that LPS induces cellular senescence in microglia. This observation aligns with previous studies demonstrating that repeated or prolonged LPS stimulation triggers microglial senescence, characterized by increased β-galactosidase activity, cell cycle arrest, and a proinflammatory phenotype (Kitchener et al., 2023[18]). Notably, pretreatment with ibrutinib at both 1 μM and 10 μM concentrations markedly reduced the number of GLB-1 positive cells induced by LPS, suggesting that ibrutinib attenuates LPS-driven microglial senescence.

The reduction of senescent cells by ibrutinib may contribute to its neuroprotective effects because senescent microglia are known to adopt dysfunctional proinflammatory states that exacerbate neurodegeneration. Our data thus provide novel evidence that ibrutinib not only modulates inflammatory responses but also mitigates the onset of cellular senescence in activated microglia, which may help preserve microglial homeostasis under inflammatory stress.

### Ibrutinib reverses LPS-induced cell cycle arrest in G0/G1 phase of microglia

**Figure 8:**
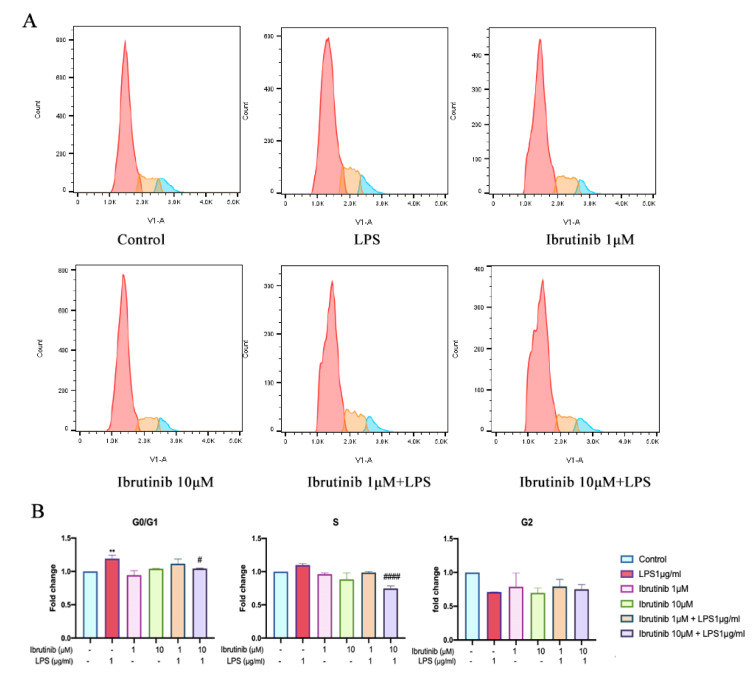
Ibrutinib suppressed LPS induced cell cycle arrest in G0/G1 phase of C8-B4 microglial cells. Data are expressed as mean ± standard deviation (SD) of three independent experiments. ^**^P < 0.01 indicate that the cell group is significantly different from control group and ^#^P<0.05, ^####^P<0.0001 indicate that the cell group is significantly different from LPS group.

Flow cytometry analysis demonstrated that exposure to LPS resulted in a significant accumulation of C8-B4 microglial cells in the G0/G1 phase of the cell cycle, indicative of LPS-induced cell cycle arrest at this checkpoint. This arrest likely reflects a cellular stress response associated with inflammation and impaired proliferative capacity of microglia. Notably, pretreatment with 10 μM ibrutinib effectively reduced the proportion of cells stalled in the G_0_/G_1_ phase, restoring cell cycle distribution toward that observed in untreated control cells (Figure 8A and 8B(Fig. 8)). These findings suggest that ibrutinib is capable of counteracting LPS-induced cell cycle arrest, thereby supporting the maintenance of normal microglial cell cycle progression under inflammatory conditions. 

### Ibrutinib inhibits the upregulation of LPS-induced inflammatory markers in C8-B4 cells 

**Figure 9:**
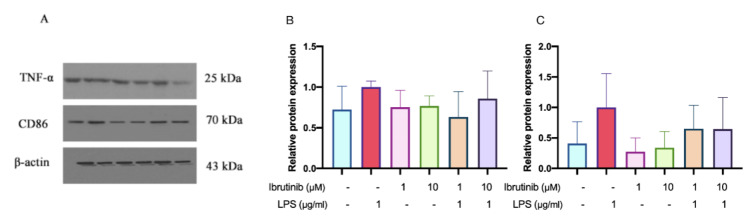
Effects of LPS and ibrutinib on the selected pro-inflammatory markers (TNF-α and CD86). C8-B4 cells were pre-treated with ibrutinib (1 and 10 μM) for 1 h and then stimulated with LPS (1 μg/mL) for additional 23 h. The levels of TNF-α and CD86 were determined by western blot analysis (A) and relative levels of TNF-α (B) and CD86 (C) were expressed as fold changes compared with control. Data are expressed as mean ± standard deviation (SD) of three independent experiments.

Stimulation of C8-B4 microglial cells with LPS led to an upward trend in the expression of pro-inflammatory markers CD86 and TNF-α; however, these increases did not reach statistical significance when compared to the control group (Figure 9(Fig. 9)). Notably, subsequent treatment with 10 μM ibrutinib resulted in a statistically significant reduction in TNF-α expression relative to the LPS group, while CD86 levels remained unchanged. These findings suggest that, under our experimental conditions, ibrutinib at a higher concentration can effectively suppress certain LPS-induced inflammatory mediators, specifically TNF-α, in microglial cells.

### Ibrutinib inhibits the LPS-induced pro-inflammatory markers in C8-B4 cells

**Figure 10:**
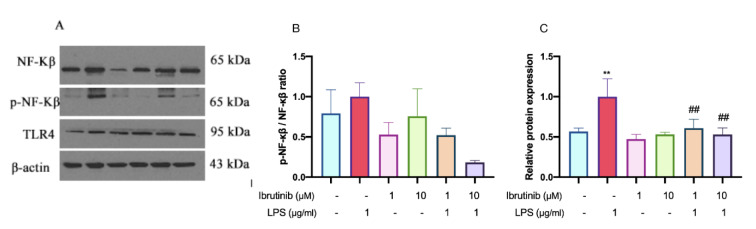
Effects of LPS and ibrutinib on TLR4 expression and the p-NF-κB/NF-κB ratio in microglia. The levels of TLR4, NF-kβ and p-NF-kβ were determined by western blot analysis (A) and relative levels of p-NF-kβ/NF-kβ (B), TLR4 (C). C8-B4 cells were pre-treated with ibrutinib (1 and 10 μM) for 1 h and then stimulated with LPS (1 μg/ml) for additional 23 h. Data are expressed as mean ± standard deviation (SD) of three independent experiments. ^*^P<0.05 and ^**^P < 0.01 indicate that the cell group is significantly different from control group and^ ##^P < 0.01 indicates that the cell group is significantly different from LPS group.

LPS stimulation of C8-B4 microglial cells led to a marked upregulation of key signaling molecules involved in inflammatory responses, specifically Toll-like receptor 4 (TLR4), nuclear factor kappa B (NF-κβ), and its active, phosphorylated form (p-NF-κβ). This increase, observed by western blot analysis, underscores activation of the classical TLR4/NF-κβ signaling pathway upon inflammatory challenge (Figure 10(Fig. 10)).

Pretreatment with ibrutinib exerted a significant modulatory effect on these molecules. Both 1 μM and 10 μM concentrations of ibrutinib notably reduced LPS-induced TLR4 expression, indicating that ibrutinib may interfere with the initial recognition of inflammatory stimuli. Moreover, 10 μM ibrutinib was particularly effective in diminishing the elevated ratio of both total NF-κβ and pNF-κβ, suggesting an attenuation of downstream inflammatory signaling and transcriptional activation.

These findings are consistent with the known role of TLR4/NF-κβ in microglial polarization and proinflammatory activation. By suppressing both the receptor and transcription factor levels after LPS stimulation, ibrutinib may help shift microglia away from an activated, proinflammatory state. Thus, our results provide evidence that ibrutinib regulates microglial activation at multiple points within the TLR4/NF-κβ axis, further supporting its potential neuroprotective effects under inflammatory conditions.

### Ibrutinib increases antioxidant capacity of LPS-treated microglia

**Figure 11:**
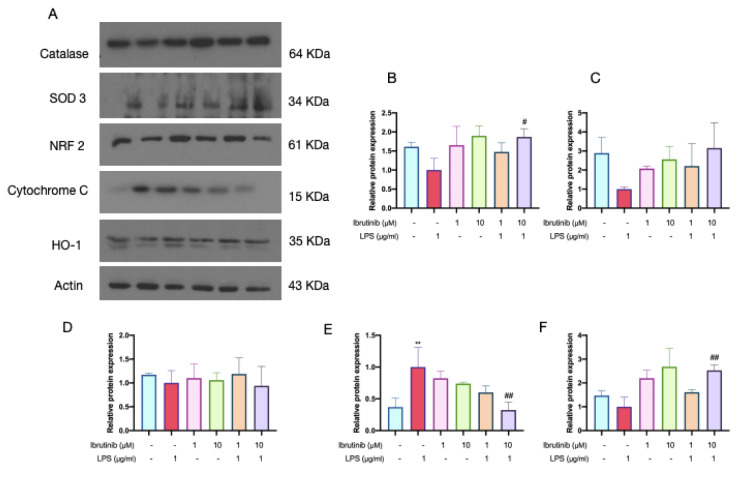
Effects of LPS and ibrutinib on activation of catalase, SOD3, Nrf 2, cytochrome c and HO-1 in microglia. The levels of catalase, SOD3, Nrf2, cytochrome c and HO-1 were determined by western blot analysis (A) and relative levels of catalase (B), SOD3 (C), Nrf 2 (D), HO-1 (E), cytochrome c (F) were expressed as fold changes compared with control. C8-B4 cells were pre-treated with ibrutinib (1 and 10 μM) for 1 h and then stimulated with LPS (1 μg/ml) for additional 23 h. Data are expressed as mean ± standard deviation (SD) of three independent experiments. ^*^P<0.05 and ^**^P < 0.01 indicate that the cell group is significantly different from control group and ^#^P<0.05, ^##^P < 0.01 indicates that the cell group is significantly different from the LPS group.

To further elucidate the antioxidant and cytoprotective effects of ibrutinib in microglial cells, we examined the expression of several key enzymes and regulatory proteins involved in oxidative stress defense pathways (Figure 11(Fig. 11)).

Treatment with 10 μM ibrutinib resulted in a significant upregulation of catalase expression (Figure 11B(Fig. 11)), suggesting that ibrutinib may enhance the cellular capacity to decompose hydrogen peroxide and thereby mitigate oxidative damage. Analysis of (SOD), another critical antioxidant enzyme, revealed that LPS exposure suppressed SOD3 expression in microglia (Figure 11C(Fig. 11)). Importantly, pretreatment with both 1 μM and 10 μM ibrutinib effectively prevented the LPS-induced downregulation of SOD3, maintaining its expression at levels comparable to untreated controls. This preservation of SOD3 expression further underscores the antioxidant protective effect of ibrutinib during inflammatory stress.

Regarding the transcription factor Nrf2, which plays a central role in regulating cellular antioxidant defense mechanisms, we observed a reduction in Nrf2 expression following LPS stimulation, although this decrease did not reach statistical significance (Figure 11D(Fig. 11)). Notably, ibrutinib pretreatment appeared to counteract the LPS-induced reduction, helping to sustain Nrf2 protein levels.

Furthermore, assessment of the downstream antioxidant enzyme HO-1, which is transcriptionally controlled by Nrf2, showed a robust and statistically significant increase in expression with 10 μM ibrutinib (Figure 11E(Fig. 11)). This pronounced induction of HO-1 suggests that ibrutinib activates the Nrf2/HO-1 axis, thereby amplifying intrinsic cellular defenses against oxidative injury.

Finally, LPS caused a significant decline in cytochrome c expression, indicative of mitochondrial dysfunction and potential initiation of apoptotic pathways. Pretreatment with 10 μM ibrutinib significantly attenuated this reduction, suggesting that ibrutinib helps in protection of mitochondrial health under inflammatory conditions (Figure 11F(Fig. 11)).

Collectively, these results demonstrate that ibrutinib reinforces the antioxidant defense network in microglia by upregulating catalase and HO-1, preserving SOD3 and Nrf2 expression, and maintaining mitochondrial cytochrome c levels. These actions may contribute to the overall neuroprotective and anti-inflammatory profile of ibrutinib observed in this study. 

## Discussion

This investigation was conducted to confirm the anti-inflammatory properties of ibrutinib and elucidate the possible involvement of the TLR4/NF-κβ and Nrf2/HO-1 pathways in LPS-induced activation of C8-B4 microglia. We have found that ibrutinib reverses the detrimental changes induced by LPS, such as oxidative stress, microglial senescence, generation of pro-inflammatory mediators, and cell cycle arrest. Therefore, our findings indicate that ibrutinib may be a promising agent in neuroinflammation-related diseases.

Primarily, we have confirmed that ibrutinib does not cause cytotoxicity up to a concentration of 25 μM. This is in line with the previous research (Nam et al., 2018[26]). We have chosen two doses (1 μM and 10 μM) and these were used in all subsequent experiments in this study. Successively, we have found that ibrutinib efficiently decreased LDH release (Figure 3(Fig. 3)), which is a marker of cell damage, in the presence of LPS. These findings suggest that ibrutinib acts as a cytoprotective agent. This was further supported by a wound healing assay, where we observed that LPS increased microglial migration, but treatment with 10 μM ibrutinib significantly diminished this effect on C8-B4 microglial cells (Figure 5(Fig. 5)). Namely, in a homeostatic state, microglia use their long extensions to continuously monitor their surroundings, while their cell bodies remain stationary. The migration of microglial cells is a key indicator of pro-inflammatory and chronic activation in the early stages of neurodegeneration (Nimmerjahn et al., 2005[28]). Ibrutinib may help maintain this homeostatic state, preventing microglia from prematurely and excessively transforming into mobile phagocytes.

LPS, the primary constituent of the outer membrane of gram-negative bacteria, serves as an inflammatory trigger capable of inducing microglial polarization (Orihuela et al., 2016[29]). In this study, we observed an increase in NO production upon stimulation of C8-B4 microglial cells with LPS and this effect was suppressed by ibrutinib in a dose-dependent manner. Moreover, the expression of NOS3, which was increased by LPS, significantly decreased with ibrutinib treatment, demonstrating its capacity to reduce oxidative stress and inhibit cellular proliferation. These findings support our migration results (Förstermann and Münzel, 2006[10]; Król and Kepinska, 2020[19]).

The overproduction of ROS by infiltrating immune cells contributes to the brain inflammation (Yeung et al., 2021[41]; Zhou et al., 2022[44]). LPS is known for inducing oxidative stress in various conditions by triggering an overproduction of ROS, particularly in models of neuroinflammation and neurodegeneration (Davalli et al., 2016[5]; Singh et al., 2019[35]; Climent et al., 2020[3]). Poor regulation of ROS signaling also leads to inducing cellular senescence. In our study, ibrutinib in both 1 μM and 10 μM doses effectively reduced intracellular ROS production in LPS-treated C8-B4 cells and significantly decreased the level of cellular senescence (Figure 7(Fig. 7)). Additionally, our findings suggest that both LPS and ibrutinib play a role in influencing the cell cycle in microglial C8-B4 cells. Namely, we explored whether LPS-induced inflammation affects the cell cycle and found that treatment with LPS, along with ibrutinib co-treatment, resulted in G_0_/G_1_ cell cycle arrest. This is probably mediated by the upregulation of cyclin-dependent kinase inhibitors such as p21(Waf1/Cip1) and GADD45α, although due to limitations we are unable perform western blot analysis on the p21 and GADD45α genes which are responsible for G1 arrest in microglia (Figure 8(Fig. 8)) (Harper et al., 1993[11]). But this cell cycle arrest is corroborated with previously reported results (Xing et al., 2022[40]). The activation of microglia and subsequent cell cycle arrest can have significant implications for neurodegenerative diseases. Excessive microglial activation may exacerbate neuroinflammation, leading to neuronal damage and loss, which is slightly decreased by ibrutinib treatment.

The reduction of oxidative stress, along with beneficial effects what follow this decrease, are mediated by the ibrutinib's capacity to upregulate antioxidant enzymes. We have found that 10 μM ibrutinib increased the expression of catalase and prevented significant decrease in SOD3 caused by LPS. This was concomitant with the elevation of the expression of Nrf2's downstream antioxidant enzyme HO-1, by 10 μM ibrutinib. Simultaneously, LPS reduced Nrf2 expression, though the reduction was not statistically significant, whereas this effect was not seen with ibrutinib pretreatment (Figure 11(Fig. 11)). These results indicate ibrutinib might be an activator of Nrf2/HO-1 pathway. Namely, Nrf2 plays a pivotal role in mediating neuroinflammation through its regulation of oxidative stress responses, such as antioxidant enzymes, like catalase and SOD3. Targeting the Nrf2/HO-1 signaling pathway presents a promising therapeutic strategy for managing neurodegenerative diseases, like multiple sclerosis and potentially other neurodegenerative diseases characterized by similar inflammatory processes.

Activated microglia release inflammatory mediators, like TNF-α. Therefore, the drugs modulating the microglial activation and reducing the release of pro-inflammatory cytokines represent a promising therapeutic strategy for neuroinflammation. The previous studies concluded that activated microglia were the main contributor of TNF-α under neuroinflammation and here our results show that ibrutinib can reduce the LPS-induced neuroinflammation (Figures 9(Fig. 9) and 10(Fig. 10)). Reduction of TNF-α in microglial cells was linked to suppression of NF-kβ's nuclear translocation, which supports previously published research (Jefferies et al., 2003[14]). Ibrutinib disrupts this critical regulatory factor involved in inflammatory cytokine production (Zusso et al., 2019[45]). However, further studies are needed to explore the underlying mechanism. 

In summary, our research demonstrates that ibrutinib can modify LPS-induced microglial activation by decreasing the levels of TLR4 and NF-κβ cytokines (Figure 10(Fig. 10)). Our data suggest that TLR4/NF-κβ pathway could be implicated in LPS-triggered microglia activation alongside the activation of Nrf2/HO-1 pathway and its downstream enzymes catalase and SOD3 (Figure 11(Fig. 11)). The modulation of these pathways by ibrutinib may facilitate the restoration of normal inflammatory levels. Despite extensive research on microglial activation and polarization, inconsistences persist in the findings, and there is limited information regarding the role of ibrutinib or BTK inhibitors in the context of oxidative stress in microglia. Additionally, while our study focused on the TLR4/ NF-κβ and Nrf2/HO-1 pathways, the complex nature of oxidative stress pathways and the impact of ibrutinib on mitochondrial proteins like cytochrome c need further exploration. Thus, future investigations are necessary to elucidate the mechanisms through which ibrutinib influences oxidative stress and various mitochondrial functions, including mitophagy. 

## Conclusion

In conclusion, ibrutinib significantly decreased the LPS-induced increase in inflammatory cytokines in C8-B4 microglial cells and modified the TLR4/NF-κβ and Nrf2/HO-1 pathway in microglia. These findings suggest that altering oxidative stress could be a promising approach for preventing and/or addressing microglial inflammation, thereby offering further evidence for its potential in treating neurodegenerative disorders. Thus, ibrutinib shows potential as a therapeutic agent for diseases related to neuroinflammation.

## Notes

Danica Michalicková and Ondrej Slanar (Institute of Pharmacology, First Faculty of Medicine, Charles University and General University Hospital, Prague, Czech Republic; E-mail: ondrej.slanar@lf1.cuni.cz) contributed equally as corresponding author.

## Declaration

### Ethics approval

Not applicable.

### Author contributions

DD performed the experiments, prepared the figures, and drafted the manuscript; AM performed the experiments and analyzed the data; DG performed the experiments, specially flow cytometry and revised the manuscript; JN, DM and OS conceptualized the project and revised and edited the manuscript. All authors have read and agreed to the published version of the manuscript.

### Funding 

This work was supported by Charles University project Cooperatio - Pharmaceutical Sciences and Charles University Grant Agency project (GA UK) 162224.

### Declaration of competing interest

The authors declare that they have no known competing financial interests or personal relationships that could have appeared to influence the work reported in this paper.

### Artificial Intelligence (AI) - Assisted Technology

No external tools were utilized throughout the entirety of this research except for the grammatical correction feature embedded in WPS Office.

### Data availability

Data will be made available on request.

## Supplementary Material

Supplementary information

## Figures and Tables

**Figure 1:**
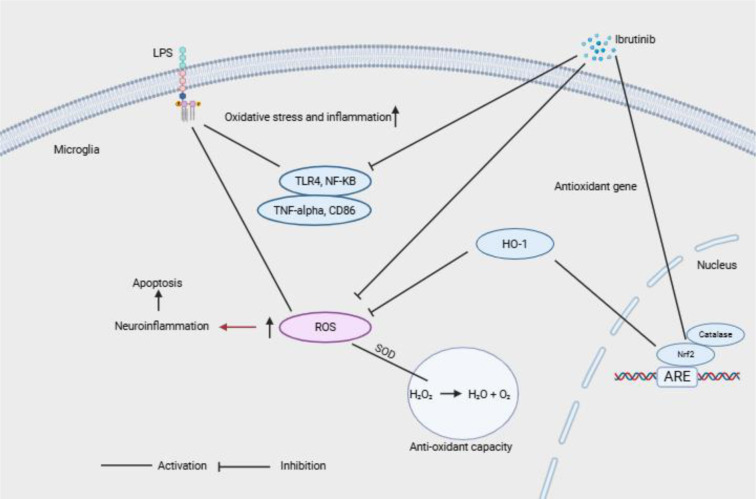
Graphical abstract

## References

[1] Acosta C, Davies A (2008). Bacterial lipopolysaccharide regulates nociceptin expression in sensory neurons. J Neurosci Res.

[2] Catorce MN, Gevorkian G (2016). LPS-induced Murine Neuroinflammation Model: Main Features and Suitability for Pre-clinical Assessment of Nutraceuticals. Curr Neuropharmacol.

[3] Climent M, Viggiani G, Chen Y-W, Coulis G, Castaldi A (2020). MicroRNA and ROS Crosstalk in Cardiac and Pulmonary Diseases. Int J Mol Sci.

[4] Das D, Ghosh A, Greco D, Michaličková D, Slanař O (2025). Bruton’s Tyrosine Kinase: A Potential Novel Target for Neurological Disorders. Physiol Res.

[5] Davalli P, Mitic T, Caporali A, Lauriola A, D’Arca D (2016). ROS, Cell Senescence, and Novel Molecular Mechanisms in Aging and Age-Related Diseases. Oxid Med Cell Longev.

[6] de Claro RA, McGinn KM, Verdun N, Lee S-L, Chiu H-J, Saber H (2015). FDA Approval: Ibrutinib for Patients with Previously Treated Mantle Cell Lymphoma and Previously Treated Chronic Lymphocytic Leukemia. Clin Cancer Res.

[7] Durafourt BA, Moore CS, Zammit DA, Johnson TA, Zaguia F, Guiot M-C (2012). Comparison of polarization properties of human adult microglia and blood-derived macrophages. Glia.

[8] Einhaus J, Pecher A-C, Asteriti E, Schmid H, Secker K-A, Duerr-Stoerzer S (2020). Inhibition of effector B cells by ibrutinib in systemic sclerosis. Arthritis Res Ther.

[9] Farrell-Dillon K, Fraser PA (2016). Pro-oxidant Nrf2 inducers: Promiscuity and protection. Vascul Pharmacol.

[10] Förstermann U, Münzel T (2006). Endothelial nitric oxide synthase in vascular disease: from marvel to menace. Circulation.

[11] Harper JW, Adami GR, Wei N, Keyomarsi K, Elledge SJ (1993). The p21 Cdk-interacting protein Cip1 is a potent inhibitor of G1 cyclin-dependent kinases. Cell.

[12] Hines DJ, Hines RM, Mulligan SJ, Macvicar BA (2009). Microglia processes block the spread of damage in the brain and require functional chloride channels. Glia.

[13] Hirsch EC, Hunot S (2009). Neuroinflammation in Parkinson’s disease: a target for neuroprotection?. Lancet Neurol.

[14] Jefferies CA, Doyle S, Brunner C, Dunne A, Brint E, Wietek C (2003). Bruton’s Tyrosine Kinase Is a Toll/Interleukin-1 Receptor Domain-binding Protein That Participates in Nuclear Factor κB Activation by Toll-like Receptor 4. J Biol Chem.

[15] Khan NM, Sandur SK, Checker R, Sharma D, Poduval TB, Sainis KB (2011). Pro-oxidants ameliorate radiation-induced apoptosis through activation of the calcium-ERK1/2-Nrf2 pathway. Free Radic Biol Med.

[16] Kim MS, Prasad V (2020). US Food and Drug Administration approvals for Bruton tyrosine kinase inhibitors in patients with chronic lymphocytic leukemia: Potential inefficiencies in trial design and evidence generation. Cancer.

[17] Kirkley KS, Popichak KA, Afzali MF, Legare ME, Tjalkens RB (2017). Microglia amplify inflammatory activation of astrocytes in manganese neurotoxicity. J Neuroinflammation.

[18] Kitchener EJA, Dundee JM, Brown GC (2023). Activated microglia release β-galactosidase that promotes inflammatory neurodegeneration. Front Aging Neurosci.

[19] Król M, Kepinska M (2020). Human Nitric Oxide Synthase-Its Functions, Polymorphisms, and Inhibitors in the Context of Inflammation, Diabetes and Cardiovascular Diseases. Int J Mol Sci.

[20] Loboda A, Damulewicz M, Pyza E, Jozkowicz A, Dulak J (2016). Role of Nrf2/HO-1 system in development, oxidative stress response and diseases: an evolutionarily conserved mechanism. Cell Mol Life Sci CMLS.

[21] Ma Q (2013). Role of nrf2 in oxidative stress and toxicity. Annu Rev Pharmacol Toxicol.

[22] Mali AS, Novotny J (2022). Opioid receptor activation suppresses the neuroinflammatory response by promoting microglial M2 polarization. Mol Cell Neurosci.

[23] Mangla A, Khare A, Vineeth V, Panday NN, Mukhopadhyay A, Ravindran B (2004). Pleiotropic consequences of Bruton tyrosine kinase deficiency in myeloid lineages lead to poor inflammatory responses. Blood.

[24] Michaličková D, Hrnčíř T, Canová NK, Slanař O (2020). Targeting Keap1/Nrf2/ARE signaling pathway in multiple sclerosis. Eur J Pharmacol.

[25] Michaličková D, Kübra Öztürk H, Hroudová J, Ľupták M, Kučera T, Hrnčíř T (2022). Edaravone attenuates disease severity of experimental auto-immune encephalomyelitis and increases gene expression of Nrf2 and HO-1. Physiol Res.

[26] Nam HY, Nam JH, Yoon G, Lee J-Y, Nam Y, Kang H-J (2018). Ibrutinib suppresses LPS-induced neuroinflammatory responses in BV2 microglial cells and wild-type mice. J Neuroinflammation.

[27] Niiro H, Clark EA (2002). Regulation of B-cell fate by antigen-receptor signals. Nat Rev Immunol.

[28] Nimmerjahn A, Kirchhoff F, Helmchen F (2005). Resting microglial cells are highly dynamic surveillants of brain parenchyma in vivo. Science.

[29] Orihuela R, McPherson CA, Harry GJ (2016). Microglial M1/M2 polarization and metabolic states. Br J Pharmacol.

[30] Paolicelli RC, Bolasco G, Pagani F, Maggi L, Scianni M, Panzanelli P (2011). , Synaptic Pruning by Microglia Is Necessary for Normal Brain Development. Science.

[31] Rip J, de Bruijn MJW, Appelman MK, Pal Singh S, Hendriks RW, Corneth OBJ (2019). Toll-Like Receptor Signaling Drives Btk-Mediated Autoimmune Disease. Front Immunol.

[32] Saijo K, Winner B, Carson CT, Collier JG, Boyer L, Rosenfeld MG (2009). A Nurr1/CoREST pathway in microglia and astrocytes protects dopaminergic neurons from inflammation-induced death. Cell.

[33] Schafer DP, Lehrman EK, Kautzman AG, Koyama R, Mardinly AR, Yamasaki R (2012). Microglia Sculpt Postnatal Neural Circuits in an Activity and Complement-Dependent Manner. Neuron.

[34] Sierra A, Encinas JM, Deudero JJP, Chancey JH, Enikolopov G, Overstreet-Wadiche LS (2010). Microglia Shape Adult Hippocampal Neurogenesis through Apoptosis-Coupled Phagocytosis. Cell Stem Cell.

[35] Singh A, Kukreti R, Saso L, Kukreti S (2019). Oxidative Stress: A Key Modulator in Neurodegenerative Diseases. Mol Basel Switz.

[36] Tan H-Y, Wang N, Li S, Hong M, Wang X, Feng Y (2016). The Reactive Oxygen Species in Macrophage Polarization: Reflecting Its Dual Role in Progression and Treatment of Human Diseases. Oxid Med Cell Longev.

[37] Tang Y, Le W (2016). Differential Roles of M1 and M2 Microglia in Neurodegenerative Diseases. Mol Neurobiol.

[38] Warren HS, Fitting C, Hoff E, Adib‐Conquy M, Beasley‐Topliffe L, Tesini B (2010). Resilience to Bacterial Infection: Difference between Species Could Be Due to Proteins in Serum. J Infect Dis.

[39] Wen T, Wang J, Shi Y, Qian H, Liu P (2021). Inhibitors targeting Bruton’s tyrosine kinase in cancers: drug development advances. Leukemia.

[40] Xing J, Fan S, Liu H, Zhang S, Li N (2022). CircZNF644 aggravates lipopolysaccharide-induced HK-2 cell impairment via the miR-140-5p/MLKL axis. J Bioenerg Biomembr.

[41] Yeung AWK, Tzvetkov NT, Georgieva MG, Ognyanov IV, Kordos K, Jóźwik A (2021). Reactive Oxygen Species and Their Impact in Neurodegenerative Diseases: Literature Landscape Analysis. Antioxid Redox Signal.

[42] Yin L, Dai Q, Jiang P, Zhu L, Dai H, Yao Z (2018). Manganese exposure facilitates microglial JAK2-STAT3 signaling and consequent secretion of TNF-a and IL-1β to promote neuronal death. Neurotoxicology.

[43] Zhang B, Yang Y, Yi J, Zhao Z, Ye R (2021). Hyperglycemia modulates M1/M2 macrophage polarization via reactive oxygen species overproduction in ligature‐induced periodontitis. J Periodontal Res.

[44] Zhou Y, Zhen Y, Wang G, Liu B (2022). Deconvoluting the Complexity of Reactive Oxygen Species (ROS) in Neurodegenerative Diseases. Front Neuroanat.

[45] Zusso M, Lunardi V, Franceschini D, Pagetta A, Lo R, Stifani S (2019). Ciprofloxacin and levofloxacin attenuate microglia inflammatory response via TLR4/NF-kB pathway. J Neuroinflammation.

