# Supplementary material for: BTK inhibitor ibrutinib reduces LPS-induced inflammation in C8-B4 microglia
Source: EXCLI J. 2025 Nov 5;24:1482–99. doi: 10.17179/excli2025-8695 (PMC12627995; doi:10.17179/excli2025-8695)
Supplement: Supplementary information [file EXCLI-24-1482-s-001.pdf]

EXCLI Journal 2025;24:1482-1499 – ISSN 1611-2156
Received: June 27, 2025, accepted: September 08, 2025, published: November 05, 2025

Supplementary information to:

Original article:

BTK INHIBITOR IBRUTINIB REDUCES LPS-INDUCED INFLAMMATION IN C8-B4 MICROGLIA

Debanjan Das[1], Akash S Mali[2,3], Denise Greco[2], Danica Michaličková[1*], Jiří Novotný[2], Ondřej Slanař[1*]

[1] Institute of Pharmacology, First Faculty of Medicine, Charles University and General University Hospital, Prague, Czech Republic
[2] Department of Physiology, Faculty of Science, Charles University, Prague, Czech Republic
[3] A. I. Virtanen Institute for Molecular Sciences, University of Eastern Finland, Kuopio, Finland

* **Corresponding authors:** Danica Michaličková, Institute of Pharmacology, First Faculty of Medicine, Charles University and General University Hospital, Prague, Czech Republic, E-mail: danica.michalickova@lf1.cuni.cz
Ondřej Slanař, Institute of Pharmacology, First Faculty of Medicine, Charles University and General University Hospital, Prague, Czech Republic, E-mail: ondrej.slanar@lf1.cuni.cz

https://dx.doi.org/10.17179/excli2025-8695

This is an Open Access article distributed under the terms of the Creative Commons Attribution License (http://creativecommons.org/licenses/by/4.0/).

This supplementary file presents the time-dependent changes in the p-NF-κB/NF-κB ratio measured at 3, 6, and 9 hours post-treatment. Raw data for the corresponding western blot experiments are included, featuring Ponceau S staining results to confirm protein loading and transfer quality. These datasets provide additional support for the results described in the main manuscript. All original data and uncropped blot images are supplied to ensure transparency and reproducibility of the findings.

EXCLI Journal 2025;24:1482-1499 – ISSN 1611-2156
Received: June 27, 2025, accepted: September 08, 2025, published: November 05, 2025

Figure S1: Time-dependent changes of p-NF-kβ/NF-kβ ratio for 3h, 6h and 9h

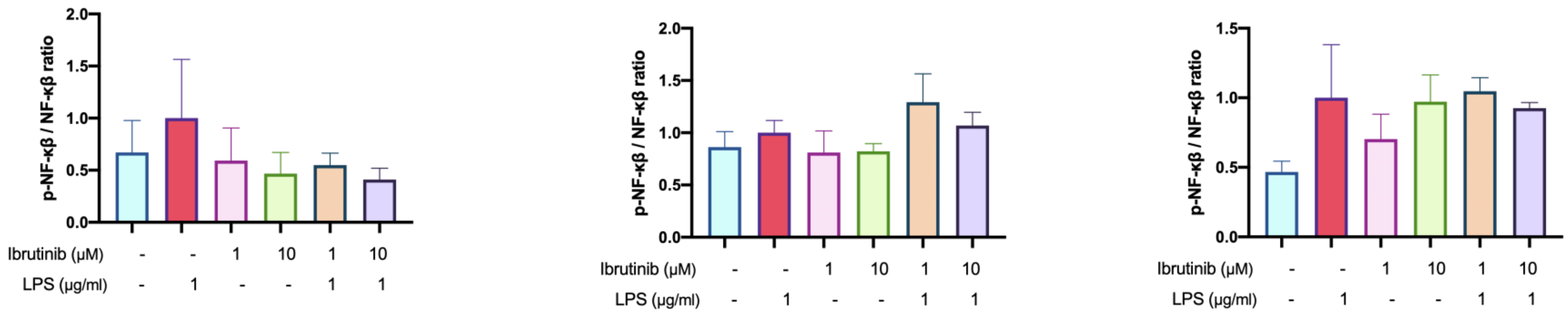

3h 6h 9h

Note: Time-dependent changes in NF-κB and phosphorylated NF-κB protein levels were assessed by western blot analysis. Cells were harvested at designated time points following treatment, and whole cell lysates were prepared for protein separation and transfer. The membranes were probed with specific antibodies to detect both total and phosphorylated forms of NF-kβ. Densitometric quantification was performed and normalized to β-actin, allowing for comparison of expression profiles over the experimental time course.

EXCLI Journal 2025;24:1482-1499 – ISSN 1611-2156
Received: June 27, 2025, accepted: September 08, 2025, published: November 05, 2025

Figure S2: Raw data of the western blot experiment with Ponceau S which is mentioned in the main manuscript

Figure 4: Example of original NOS 3 140 kDa (SC-654) western blot for three repeats with ponceau s stain

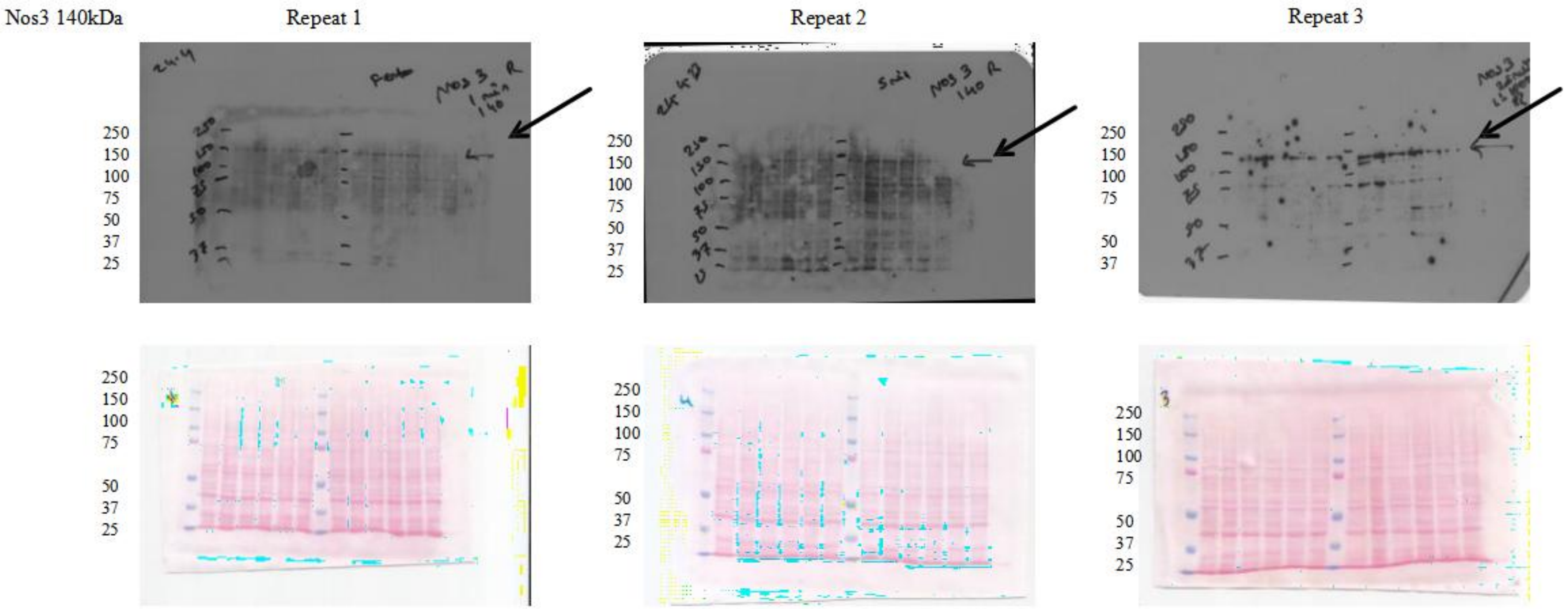

Note: The molecular weights shown on the left side are given for better understanding of molecular weight written by hand in the blot. Please note that, due to technical reasons, the corresponding values may not be fully in the same row. Three different generations of cells were used for the three repetitions, and the black arrow identifies the band used for statistical analysis.

EXCLI Journal 2025;24:1482-1499 – ISSN 1611-2156
Received: June 27, 2025, accepted: September 08, 2025, published: November 05, 2025

Figure 4: Example of original actin (SC-47778) western blot for three repeats with ponceau s stain

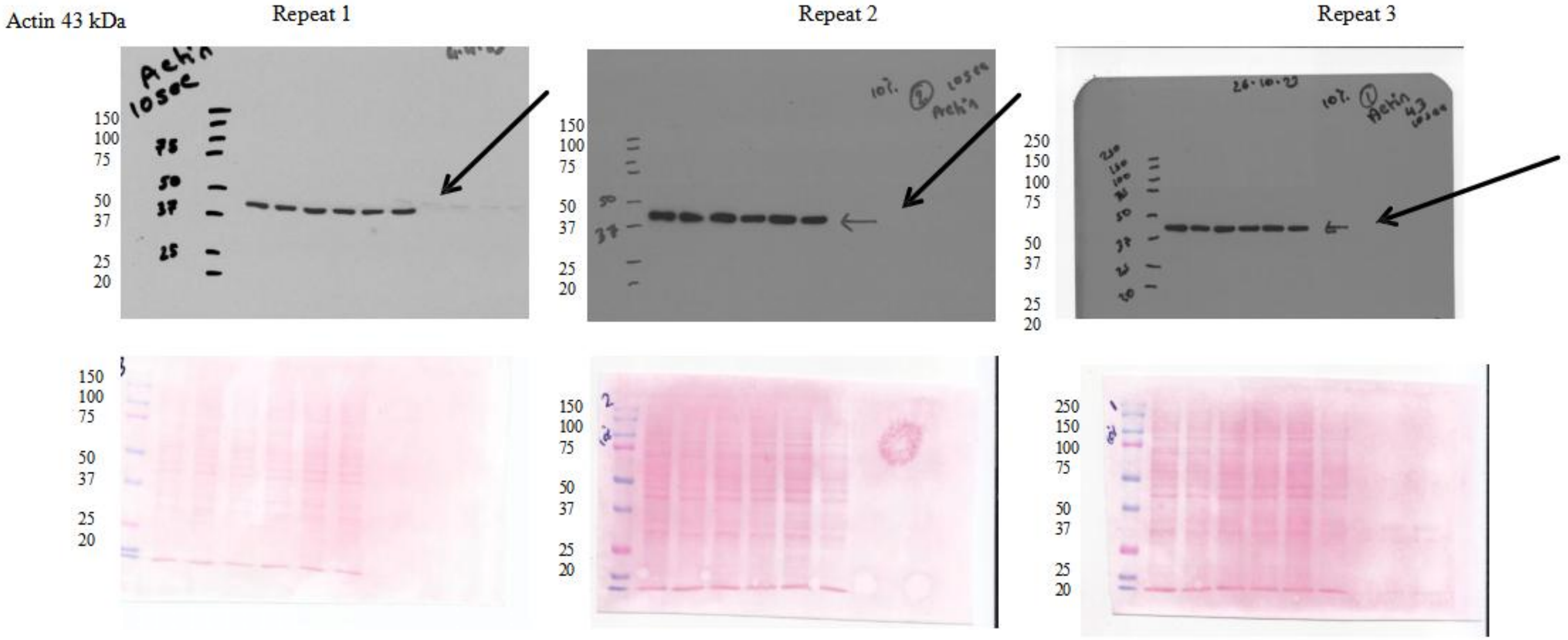

Note: The molecular weights shown on the left side are given for better understanding of molecular weight written by hand in the blot. Please note that, due to technical reasons, the corresponding values may not be fully in the same row. Three different generations of cells were used for the three repetitions, and the black arrow identifies the band used for statistical analysis.

EXCLI Journal 2025;24:1482-1499 – ISSN 1611-2156
Received: June 27, 2025, accepted: September 08, 2025, published: November 05, 2025

Figure 9: Example of original TNF-α (SC-52746) western blot for three repeats with ponceau s stain

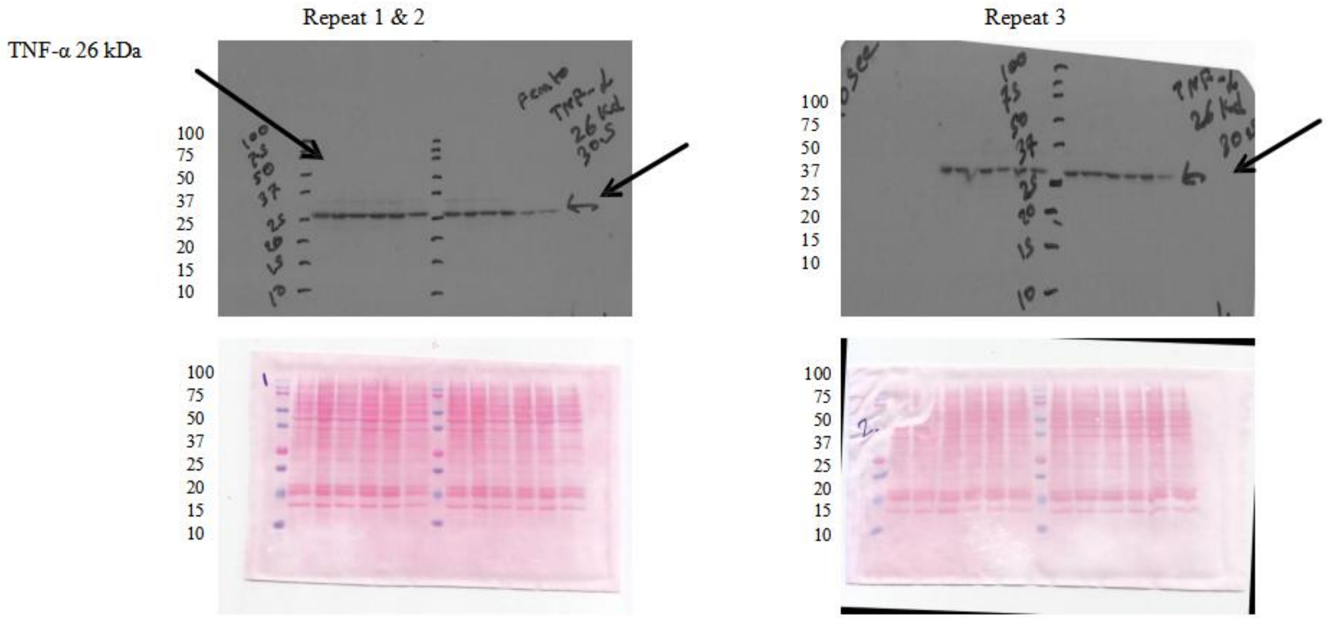

Note: The molecular weights shown on the left side are given for better understanding of molecular weight written by hand in the blot. Please note that, due to technical reasons, the corresponding values may not be fully in the same row. Three different generations of cells were used for the three repetitions, and the black arrow identifies the band used for statistical analysis.

EXCLI Journal 2025;24:1482-1499 – ISSN 1611-2156
Received: June 27, 2025, accepted: September 08, 2025, published: November 05, 2025

Figure 9: Example of original CD86/B7-2 (SC-28347) western blot for three repeats with ponceau s stain

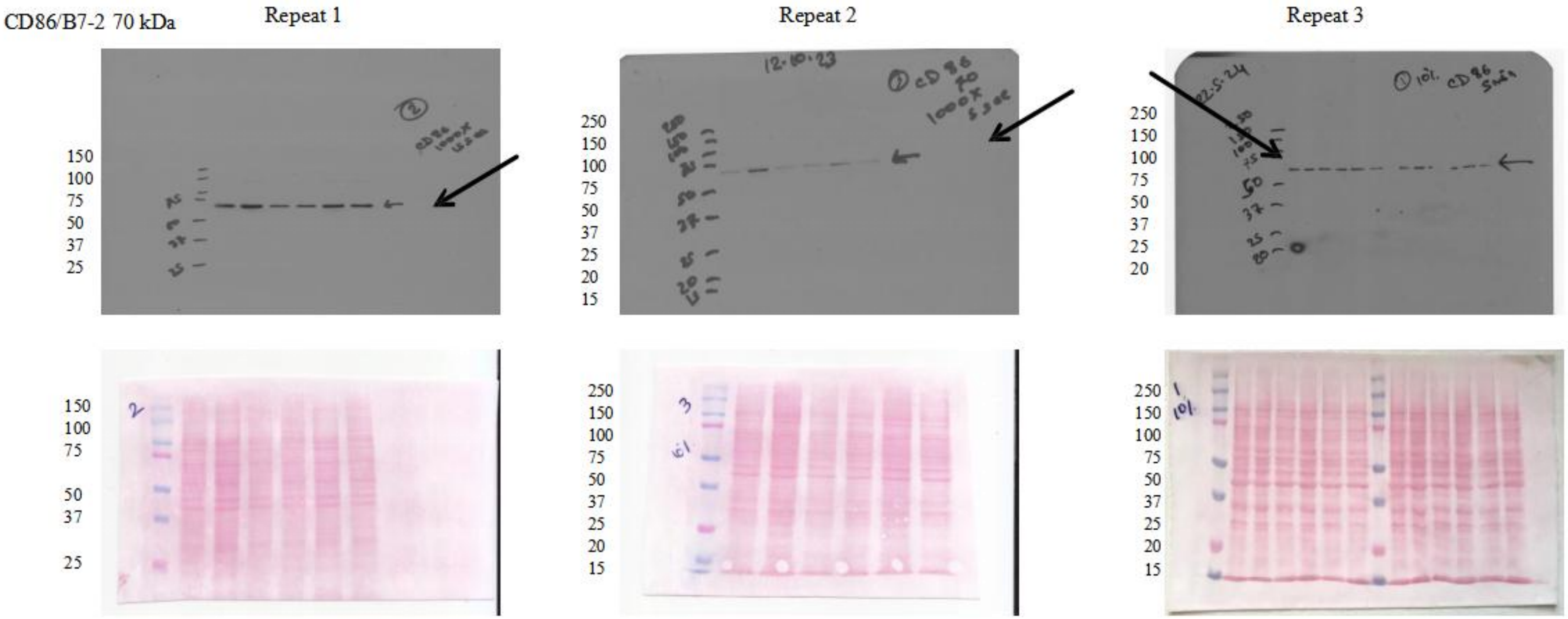

Note: The molecular weights shown on the left side are given for better understanding of molecular weight written by hand in the blot. Please note that, due to technical reasons, the corresponding values may not be fully in the same row. Three different generations of cells were used for the three repetitions, and the black arrow identifies the band used for statistical analysis.

EXCLI Journal 2025;24:1482-1499 – ISSN 1611-2156
Received: June 27, 2025, accepted: September 08, 2025, published: November 05, 2025

Figure 10: Example of original NF-kβ (SC-514451) western blot for three repeats with ponceau s stain

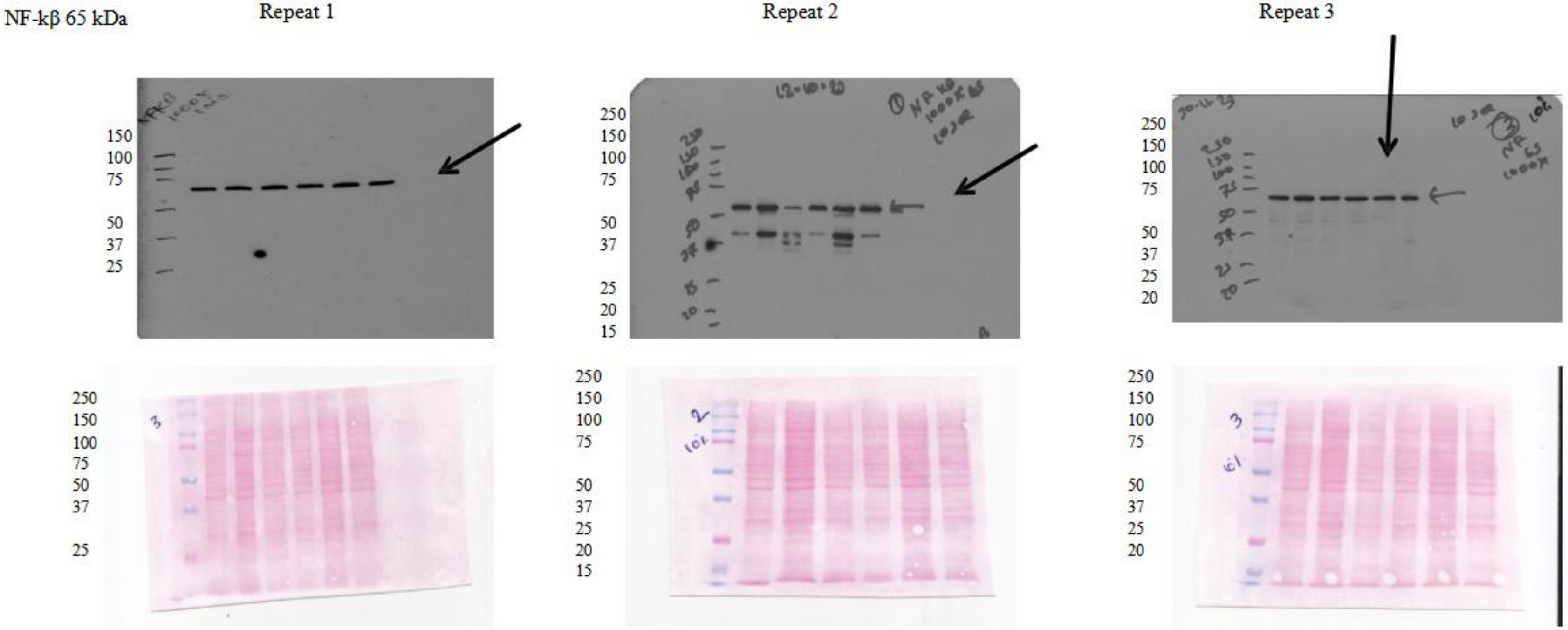

Note: The molecular weights shown on the left side are given for better understanding of molecular weight written by hand in the blot. Please note that, due to technical reasons, the corresponding values may not be fully in the same row. Three different generations of cells were used for the three repetitions, and the black arrow identifies the band used for statistical analysis.

EXCLI Journal 2025;24:1482-1499 – ISSN 1611-2156
Received: June 27, 2025, accepted: September 08, 2025, published: November 05, 2025

Figure 10: Example of original p-NF-kβ (SC-135769) western blot for three repeats with ponceau s stain

p-NF-kβ 65 kDa — Repeat 1 — Repeat 2 — Repeat 3

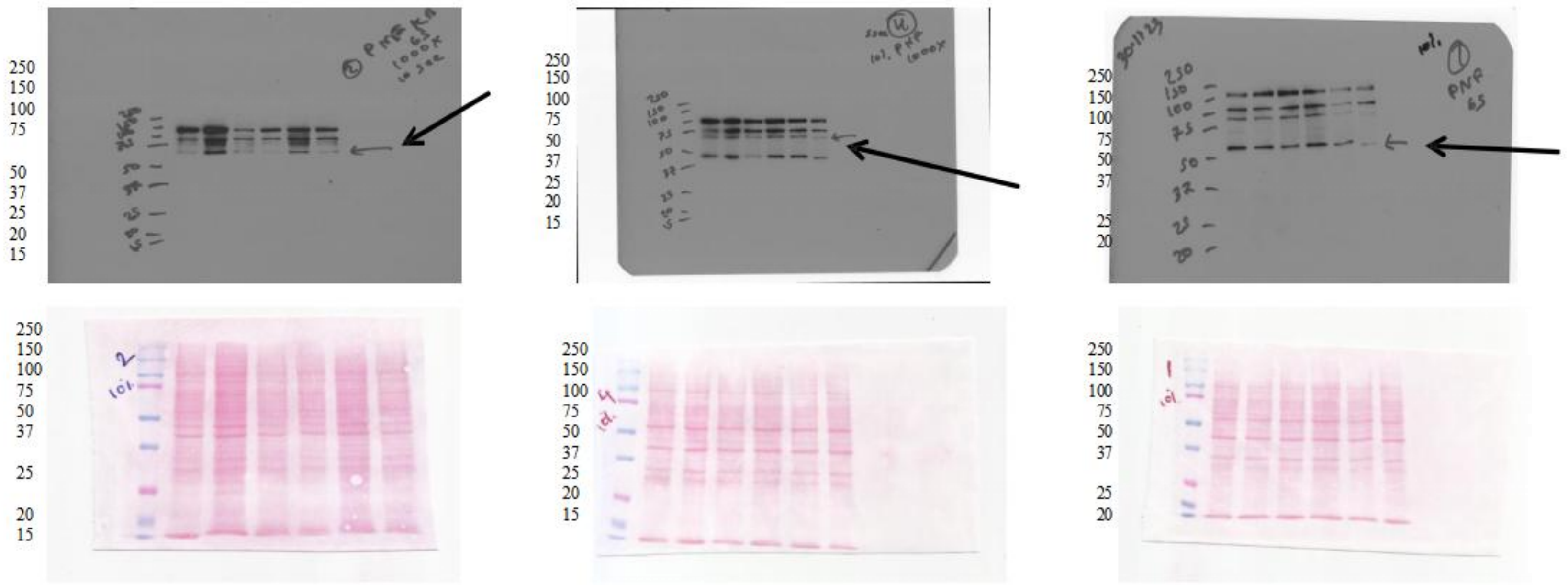

Note: The molecular weights shown on the left side are given for better understanding of molecular weight written by hand in the blot. Please note that, due to technical reasons, the corresponding values may not be fully in the same row. Three different generations of cells were used for the three repetitions, and the black arrow identifies the band used for statistical analysis.

EXCLI Journal 2025;24:1482-1499 – ISSN 1611-2156
Received: June 27, 2025, accepted: September 08, 2025, published: November 05, 2025

Figure 10: Example of original TLR4 (SC-52746) western blot for three repeats with ponceau s stain

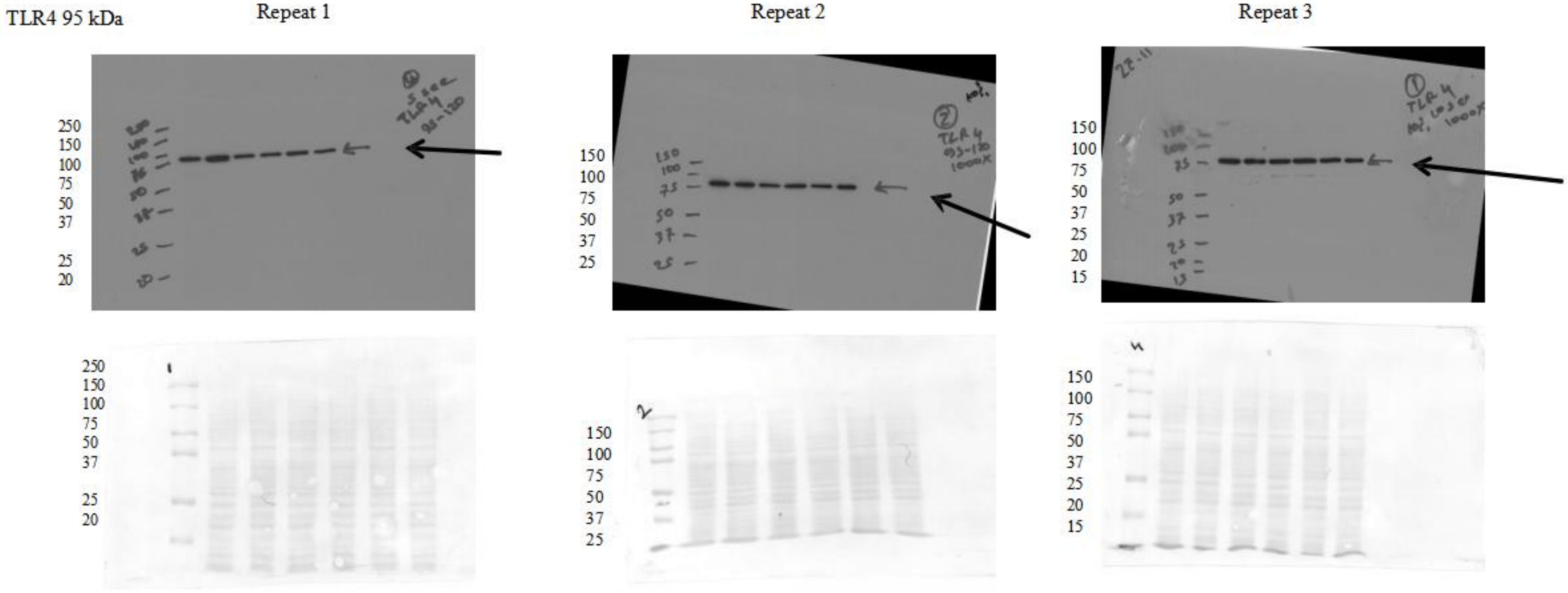

Note: The molecular weights shown on the left side are given for better understanding of molecular weight written by hand in the blot. Please note that, due to technical reasons, the corresponding values may not be fully in the same row. Three different generations of cells were used for the three repetitions, and the black arrow identifies the band used for statistical analysis.

EXCLI Journal 2025;24:1482-1499 – ISSN 1611-2156
Received: June 27, 2025, accepted: September 08, 2025, published: November 05, 2025

Figure 11: Example of original catalase (SC-271803) western blot for three repeats with ponceau s stain

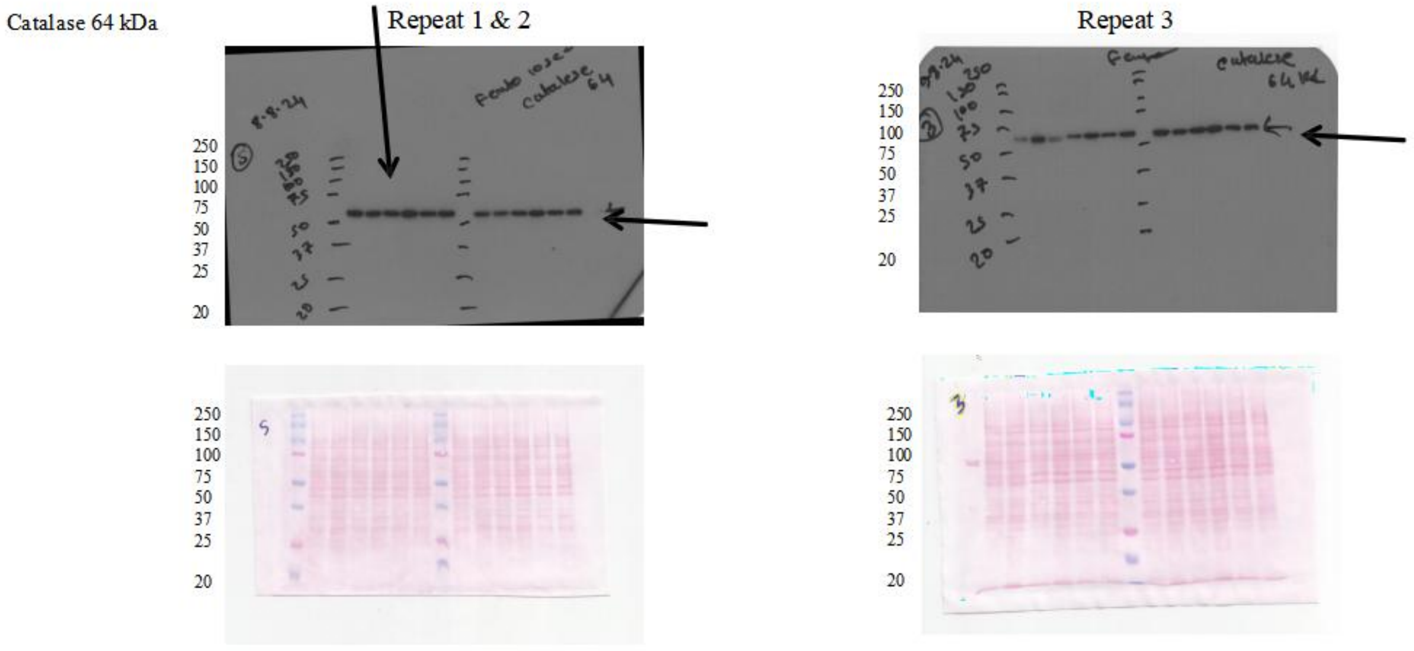

Note: The molecular weights shown on the left side are given for better understanding of molecular weight written by hand in the blot. Please note that, due to technical reasons, the corresponding values may not be fully in the same row. Three different generations of cells were used for the three repetitions, and the black arrow identifies the band used for statistical analysis.

EXCLI Journal 2025;24:1482-1499 – ISSN 1611-2156
Received: June 27, 2025, accepted: September 08, 2025, published: November 05, 2025

Figure 11: Example of original |SOD3 (A6984) western blot for three repeats with ponceau s stain

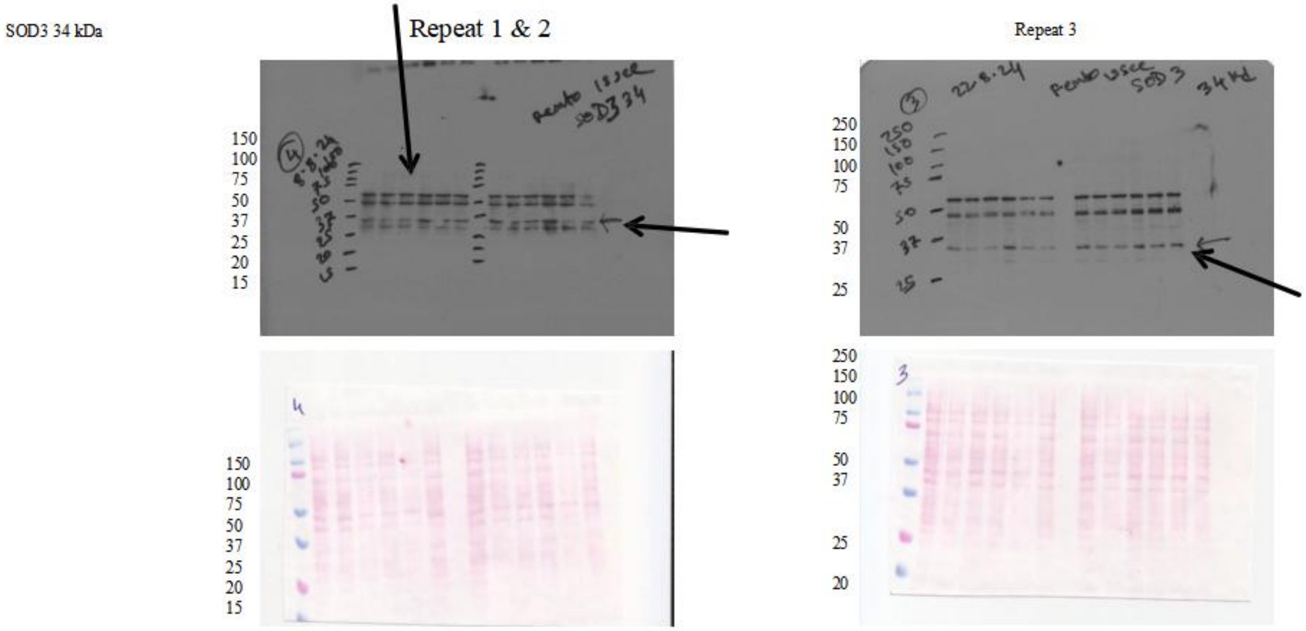

Note: The molecular weights shown on the left side are given for better understanding of molecular weight written by hand in the blot. Please note that, due to technical reasons, the corresponding values may not be fully in the same row. Three different generations of cells were used for the three repetitions, and the black arrow identifies the band used for statistical analysis.

EXCLI Journal 2025;24:1482-1499 – ISSN 1611-2156
Received: June 27, 2025, accepted: September 08, 2025, published: November 05, 2025

Figure 11: Example of original NRF2 (SC-722) western blot for three repeats with ponceau s stain

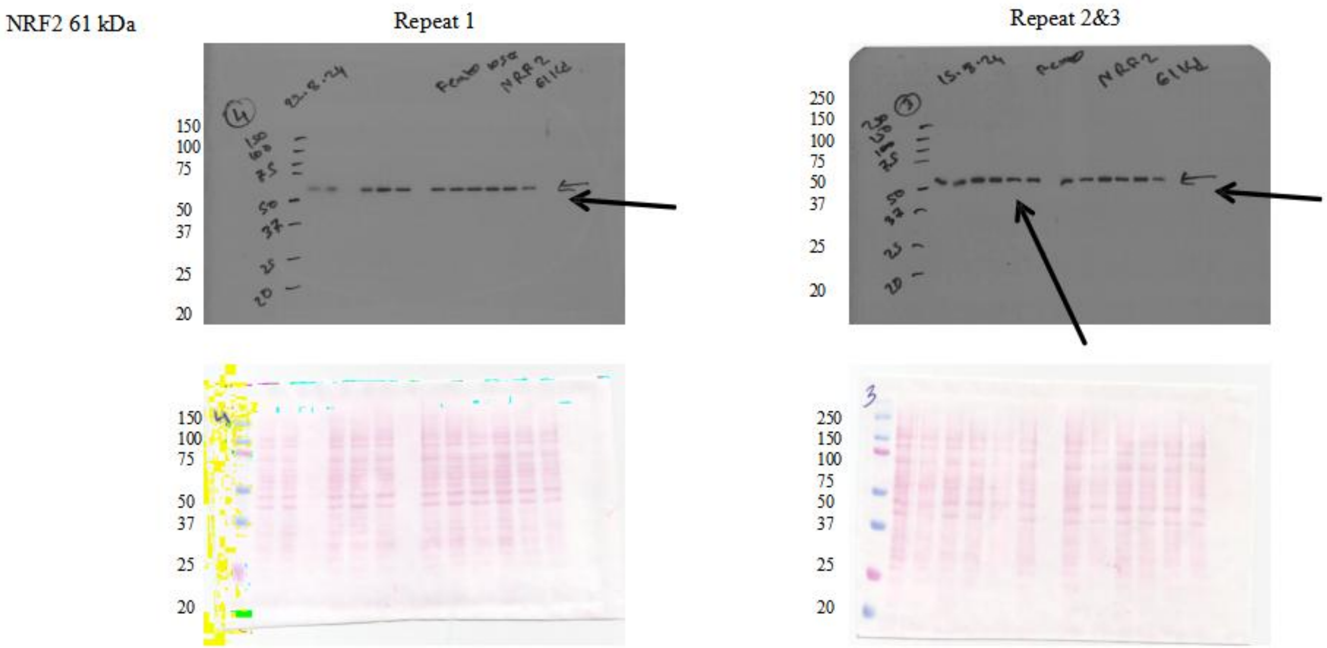

Note: The molecular weights shown on the left side are given for better understanding of molecular weight written by hand in the blot. Please note that, due to technical reasons, the corresponding values may not be fully in the same row. Three different generations of cells were used for the three repetitions, and the black arrow identifies the band used for statistical analysis.

EXCLI Journal 2025;24:1482-1499 – ISSN 1611-2156
Received: June 27, 2025, accepted: September 08, 2025, published: November 05, 2025

Figure 11: Example of original HO-1(A19062) western blot for three repeats with ponceau s stain

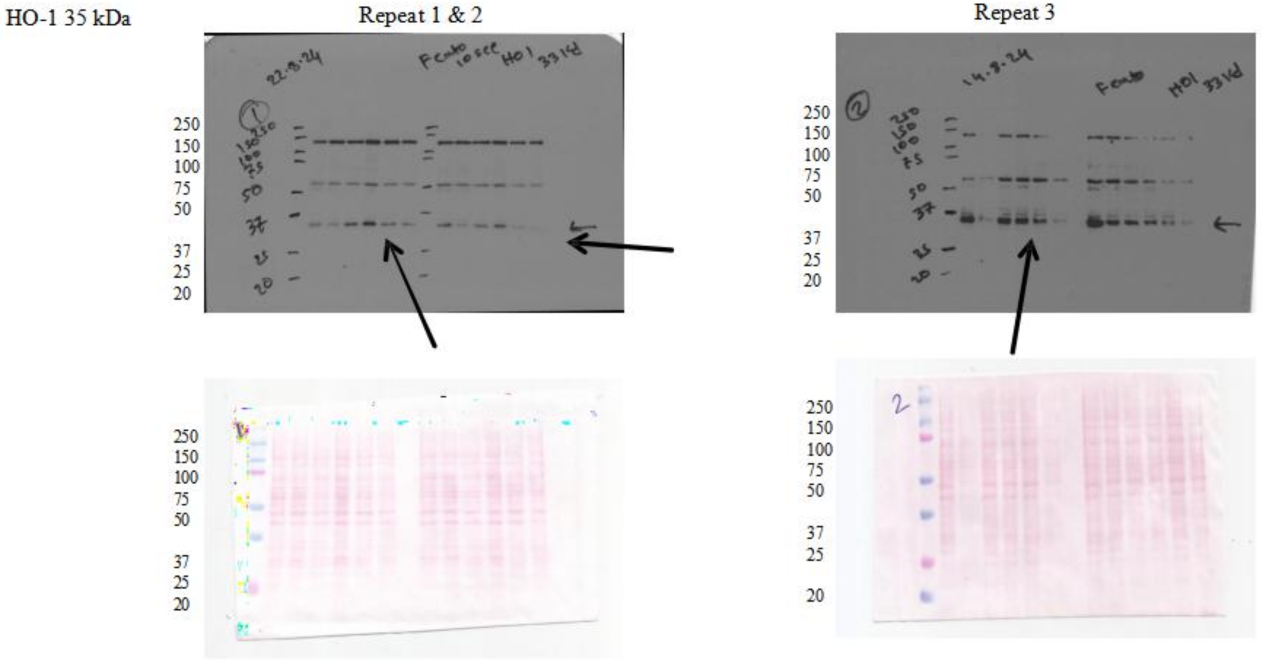

Note: The molecular weights shown on the left side are given for better understanding of molecular weight written by hand in the blot. Please note that, due to technical reasons, the corresponding values may not be fully in the same row. Three different generations of cells were used for the three repetitions, and the black arrow identifies the band used for statistical analysis.

Received: June 27, 2025, accepted: September 08, 2025, published: November 05, 2025

Figure 11: Example of original cytochrome-c (SC-13156) western blot for three repeats with ponceau s stain

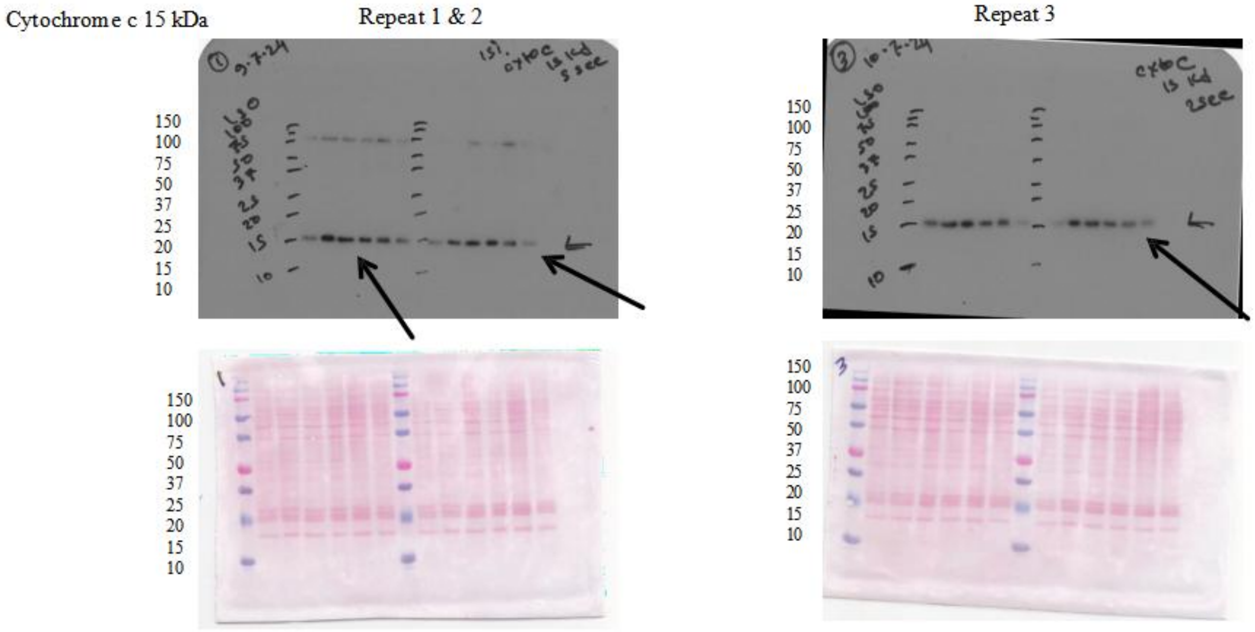

Note: The molecular weights shown on the left side are given for better understanding of molecular weight written by hand in the blot. Please note that, due to technical reasons, the corresponding values may not be fully in the same row. Three different generations of cells were used for the three repetitions, and the black arrow identifies the band used for statistical analysis.

EXCLI Journal 2025;24:1482-1499 – ISSN 1611-2156
Received: June 27, 2025, accepted: September 08, 2025, published: November 05, 2025

Figure 11: Example of original actin (SC-47778) western blot for three repeats with ponceau s stain

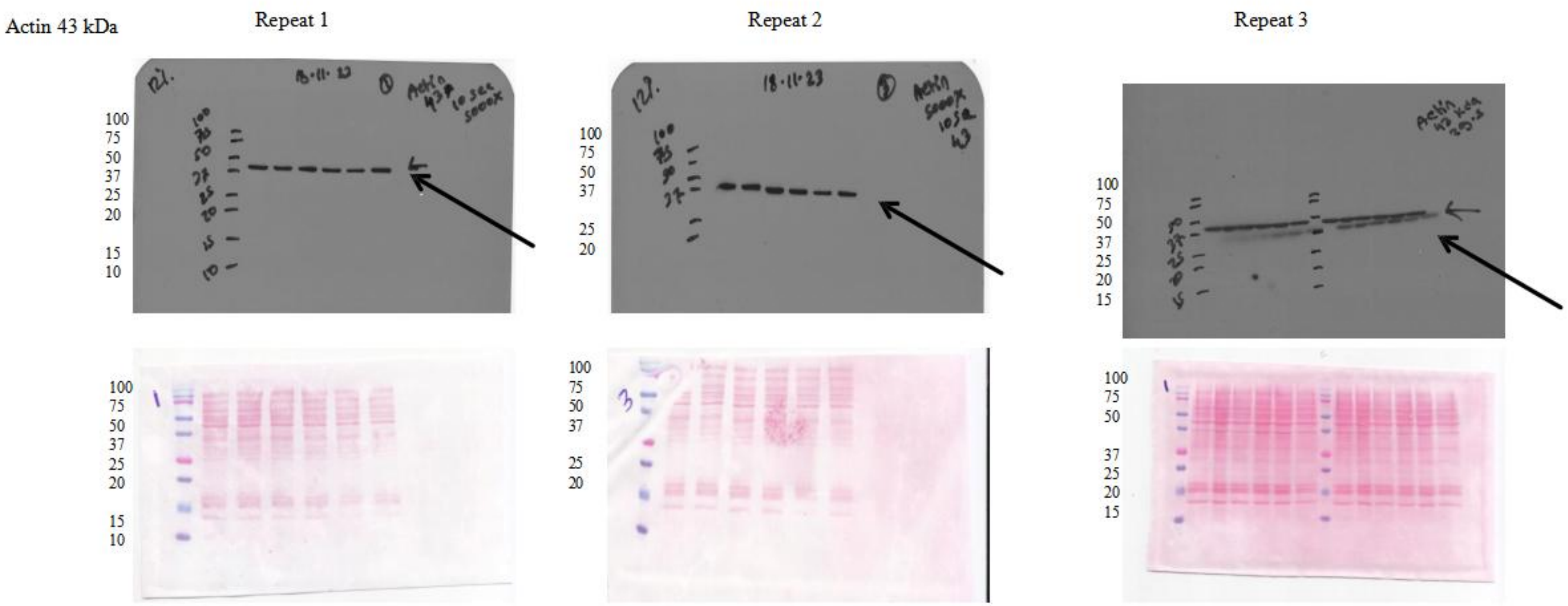

Note: The molecular weights shown on the left side are given for better understanding of molecular weight written by hand in the blot. Please note that, due to technical reasons, the corresponding values may not be fully in the same row. Three different generations of cells were used for the three repetitions, and the black arrow identifies the band used for statistical analysis.
